# Managing negative linear compressibility and thermal expansion through steric hindrance: a case study of 1,2-bis­(4′-pyridyl)­ethane cocrystals

**DOI:** 10.1107/S2052252524011734

**Published:** 2025-01-01

**Authors:** Ewa Patyk-Kaźmierczak, Kornelia Szymańska, Michał Kaźmierczak

**Affiliations:** a Facuty of Chemistry Adam Mickiewicz University in Poznań Uniwersytetu Poznańskiego 8 Poznań61-614 Poland; Sun Yat-Sen University, China

**Keywords:** negative linear compressibility, NLC, cocrystals, negative thermal expansion, NTE, crystal engineering, multicomponent crystals

## Abstract

Multicomponent crystals have great scientific potential because of their amenability to crystal engineering in terms of composition and structure, and hence their properties can be easily modified. More and more research areas are employing the design of multicomponent materials to improve the known or induce novel physicochemical properties of crystals, and recently they have been explored as materials with abnormal pressure behaviour. The cocrystal of 1,2-bis­(4′-pyridyl)­ethane and fumaric acid (ETYFUM) exhibits a negative linear compressibility behaviour comparable to that of framework and metal-containing materials, but overcomes many of their deficiencies restricting their use. Herein ETYFUM was investigated at low temperature to reveal negative thermal expansion behaviour. Additionally, a cocrystal isostructural with ETYFUM, based on 1,2-bis­(4′-pyridyl)­ethane and succinic acid (ETYSUC), was exposed to high pressure and low temperature, showing that its behaviour is similar in nature to that of ETYFUM, but significantly differs in the magnitude of both effects. It was revealed that the minor structural difference between the acid molecules does not significantly affect the packing under ambient conditions, but has far-reaching consequences when it comes to the deformation of the structure when exposed to external stimuli.

## Abstract 2

The negative linear compressibility and negative thermal expansion behaviours of two isostructural 1:1 cocrystals of 1,2-bis­(4′-pyridyl)­ethane with fumaric and succinic acids were revealed and compared, showing surprisingly strong ramifications of a small steric hindrance on the magnitude of both effects.

## 1. Introduction

Multicomponent organic crystals have attracted a lot of interest as their composition, molecular aggregation and intermolecular interactions can be tuned and their synthesis is cost efficient (Ding *et al.*, 2024 ▸). They can exhibit fluorescence (Li *et al.*, 2016 ▸; Dai *et al.*, 2024 ▸), phospho­rescence (Zhou *et al.*, 2020 ▸; Qu *et al.*, 2023 ▸), light-induced dynamic movement (Li *et al.*, 2020 ▸), static photonic properties (Li *et al.*, 2020 ▸) and temperature- and pressure-induced proton transfer (Bhunia *et al.*, 2010 ▸; Jones *et al.*, 2014 ▸; Patyk-Kaźmierczak *et al.*, 2024 ▸), or can be means to improve physicochemical properties of active pharmaceutical ingredients (Duggirala *et al.*, 2015 ▸; Bolla *et al.*, 2022 ▸). Recently, a molecular cocrystal was discovered to exhibit significant negative linear compressibility (NLC) behaviour of a magnitude previously associated with framework materials. This 1:1 cocrystal composed of 1,2-bis­(4′-pyridyl)­ethane (ETY) and fumaric acid (FUM), herein referred to as ETYFUM (Patyk-Kaźmierczak & Kaźmierczak, 2024 ▸), has a median compressibility of −24 (1) TPa^−1^ in the NLC direction and a compressibility capacity (Cairns & Goodwin, 2015 ▸), χ_K_, equal to 6.8% (for the 0.1 MPa to 3.58 GPa pressure range). Its main advantage is its facile and environmentally friendly synthesis and the recyclability of the building blocks.

Negative compressibility behaviour is unusual, as it is expected that a crystal will respond to an increase in pressure by decreasing its dimensions. However, in rare cases, abnormal behaviour can be observed when a material expands along one (NLC) or two (negative area compressibility, NAC) principal axes (Baughman *et al.*, 1998 ▸; Cairns & Goodwin, 2015 ▸). However, to make such a process thermodynamically possible, sufficient compression must take place along the remaining principal axes/axis to achieve overall volume reduction (Baughman *et al.*, 1998 ▸; Cairns & Goodwin, 2015 ▸). On the other hand, there is no such limitation when it comes to negative thermal expansion (NTE), where linear or volumetric contraction can occur on heating (Miller *et al.*, 2009 ▸). Materials exhibiting any of the above-mentioned behaviours have many possible applications. The ability to expand under pressure makes NLC and NAC materials applicable as optical sensors or in telecommunication systems required to function under high pressure (Baughman *et al.*, 1998 ▸). Meanwhile, NTE materials can be used to compensate for the positive thermal expansion (PTE) of other materials (Takenaka, 2012 ▸).

NTE materials have been intensively studied since the mid 1990s, when the NTE behaviour of a variety of materials was explained in relation to their crystal structures (Korthuis *et al.*, 1995 ▸; Sleight, 1995 ▸; Mary *et al.*, 1996 ▸; Evans *et al.*, 1997 ▸; Lind, 2012 ▸). There are many reports of inorganic and inorganic–organic hybrid materials exhibiting NTE along one or more principal axes; however, the literature on organic NTE crystals is comparably scarce. A study of the Cambridge Structural Database from 2021 (Lee & Dumitrescu, 2021 ▸) has revealed that 37% of the deposited structures investigated at various temperatures exhibit NTE (34% axial NTE and 3% volumetric NTE), which is more than was believed. The largest NTE values found in the study reached *ca* −350 and −200 MK^−1^, but such cases can be considered outliers. More commonly, uniaxial NTE is in the range of −100 MK^−1^ (Lee & Dumitrescu, 2021 ▸).

When it comes to NLC materials, reports of significant NLC are scarce, with the most negative median compressibility usually associated with framework materials. The most impressive cases are as follows: Ag_3_[Co(CN)_6_] Phase I [*K*_NLC_ = −76 (9) TPa^−1^; Δ*p* = 0–0.19 GPa (Goodwin *et al.*, 2008 ▸)], InH(BCD)_2_ [*K*_NLC_ = −62.4 TPa^−1^; Δ*p* = 0–0.53 GPa (Zeng *et al.*, 2017 ▸)] and Zn[Au(CN)_2_]_2_ Phase I [*K*_NLC_ = −42 (5) TPa^−1^; Δ*p* = 0–1.8 GPa (Cairns *et al.*, 2013 ▸)]. Recently, density functional theory was used to uncover a massive NLC for the 3D covalent organic framework NPN-3 [*K*_NLC_ = −42.04 TPa^−1^; Δ*p* = 0–0.9 GPa (Erkartal, 2024 ▸)].

It has been shown that some topological motifs show clear predisposition to exhibit NLC (Cairns & Goodwin, 2015 ▸). Hence, a search of novel NLC materials often focuses on crystals with structures utilizing such motifs, one of which is the wine rack. There are a number of NLC crystals of wine-rack structures reported in the literature, with some of the most recent and interesting cases including the already discussed ETYFUM (Patyk-Kaźmierczak & Kaźmierczak, 2024 ▸), MCF-34 (Zeng *et al.*, 2020 ▸), Eu[Ag(CN)_2_]_3_·3H_2_O (Liu *et al.*, 2024 ▸) and [C(NH_2_)_3_]Er(HCO_2_)_2_(C_2_O_4_) (Hitchings *et al.*, 2024 ▸). MCF-34 is a metal–organic framework with two distinctive wine-rack units oriented in four ways in the crystal structure, and is the first case of a multitype wine-rack material investigated in the context of NLC. Its NLC is also significant, with *K*_NLC_ = −47.3 (2) TPa^−1^ in the 0–0.53 GPa range. Meanwhile, Eu[Ag(CN)_2_]_3_·3H_2_O is a framework material that exhibits NLC over a very wide pressure range, 0–8.2 GPa [*K*_NLC_ = −4.2 (1) TPa^−1^]. Lastly, [C(NH_2_)_3_]Er(HCO_2_)_2_(C_2_O_4_) is the first reported case of a hybrid perovskite that exhibits NLC even from ambient pressure [*K*_NLC_ = −10.1 (7) TPa^−1^ for the 0–2.63 (10) GPa range].

The NAC, NLC and NTE associated with changes along the orthogonal principal axes are easily monitored for materials of orthogonal crystal systems (orthorhombic, tetragonal and cubic). In the case of the remaining crystal systems, NLC, NAC and NTE can still be observed in the abnormal changes of the unit-cell parameters; however, to determine the magnitude and direction of each effect, a calculation of the strain along the principal axes is necessary. For instance, in the case of ETYFUM of a monoclinic system, two unit-cell parameters increase on compression although negative compressibility occurs only along one principal axis (Patyk-Kaźmierczak & Kaźmierczak, 2024 ▸).

In this work, we present the case of a cocrystal (ETYSUC) of 1,2-bis­(4′-pyridyl)­ethane (ETY) and succinic acid (SUC), the NLC behaviour of which is masked by the decrease of all three unit-cell parameters. Under ambient conditions ETYSUC is isostructural with ETYFUM, however, its response to compression is remarkably different. Finally, the NTEs of ETYSUC and ETYFUM are investigated and compared. The difference in temperature and pressure behaviour of ETYSUC and ETYFUM is rationalized in terms of the subtle structural dissimilarities between SUC and FUM molecules.

## 2. Experimental

### 2.1. Cocrystal synthesis

ETYSUC and ETYFUM were synthesized by dissolving 1,2-bis­(4′-pyridyl)­ethane (ETY) and the respective acid (succinic acid – SUC, or fumaric acid – FUM) in a 1:1 molar ratio in hot methanol and leaving the solutions for slow evaporation at room temperature or by solvent-assisted ball milling (see the supporting information for additional details).

### 2.2. X-ray diffraction experiments

Single-crystal X-ray diffraction experiments were performed using a four-circle X-ray diffractometer equipped with a copper or molybdenum X-ray tube. In all cases, *CrysAlisPro* (Rigaku Oxford Diffraction, 2020 ▸, 2022 ▸) was used for data collection, **UB**-matrix determination, data reduction and absorption correction. High-pressure conditions were provided by mounting the sample crystal in a Merrill–Bassett (Merrill & Bassett, 1974 ▸) diamond anvil cell (Fig. S1 of the supporting information). For low-temperature experiments (Figs. S2–S5), a nitrogen-flow attachment from Oxford Cryosystems was used. Crystal structure determination was performed using *ShelXS* or *ShelXT* (Sheldrick, 2008 ▸, 2015*b* ▸), while refinement was performed using *ShelXL* (Sheldrick, 2015*a* ▸), with the three programs implemented in *Olex2* (Dolomanov *et al.*, 2009 ▸) as an interface. The crystallographic details for all structures are listed in Tables S1–S16 of the supporting information. Powder X-ray diffraction experiments were performed for ETYSUC crystals obtained from ball-milling synthesis, using the Bruker D8 Advance diffractometer (equipped with a copper X-ray tube). Details on X-ray diffraction experiments and data treatment are provided in the supporting information.

### 2.3. Principal axis strain and linear coefficient of thermal expansion calculations

The principal axis strain and the linear coefficients of thermal expansion were calculated using the *PASCal* program (Cliffe & Goodwin, 2012 ▸; Lertkiattrakul *et al.*, 2023 ▸) available at https://www.pascalapp.co.uk/. For details, see the supporting information.

## 3. Results and discussion

ETYSUC crystallizes in the monoclinic crystal system, space group *I*2/*a* [originally reported in space group *C*2/*c* (Braga *et al.*, 2010 ▸) but here a non-standard setting was selected to ensure a β angle value closer to 90°]. As mentioned previously, under ambient conditions, it is isostructural with ETYFUM. The only difference between the two structures lies in the structure of the acid molecules because FUM is an unsaturated analogue of SUC. Therefore, it is evident that the difference in the hybridization of α-carbon atoms and the presence of two additional hydrogen atoms in the SUC molecule does not significantly affect the preference for the aggregation of the coformers in the cocrystal. However, this seemingly insignificant difference in molecular structures is behind the drastically different pressure and temperature behaviour of ETYFUM and ETYSUC.

### 3.1. Pressure-induced phase transition in ETYSUC

**Figure 1:**
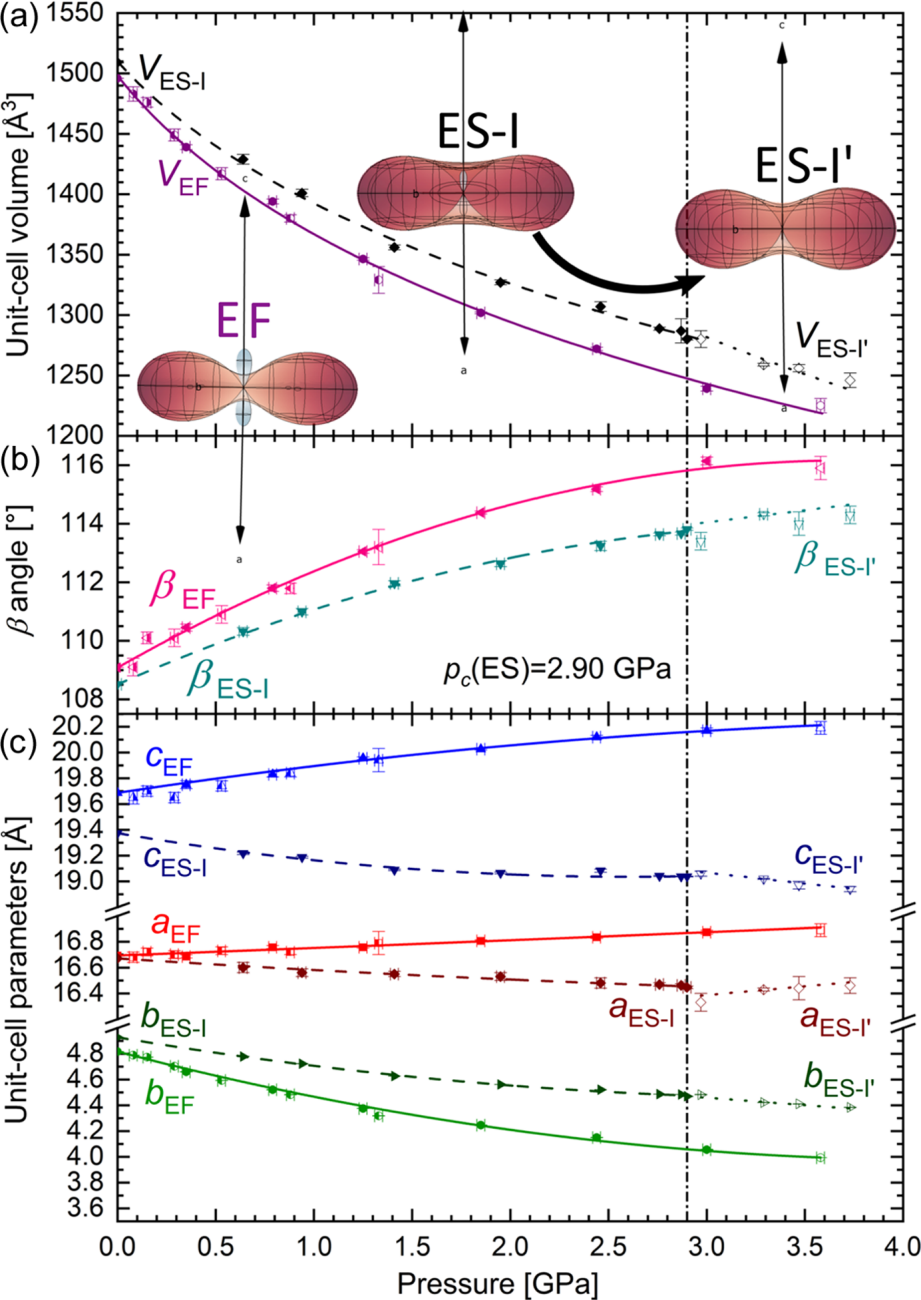
Pressure dependence of the (*a*) unit-cell volume, (*b*) β angle and (*c*) unit-cell parameters for ETYFUM (solid lines) and ETYSUC (dashed and dotted lines). For ETYFUM, various symbols mark different sample crystals investigated under pressure as done in the original work (Patyk-Kaźmierczak & Kaźmierczak, 2024 ▸). For ETYSUC, the full symbols and dashed lines are for Phase I, and the empty symbols and dotted lines are for Phase I′. The lower subscripts EF, ES-I and ES-I′ stand for ETYFUM, ETYSUC I and ETYSUC I′, respectively. The vertical dashed–dotted line marks the transition pressure (*p*_c_) for the crystal of ETYSUC. Solid and dashed lines in (*b*) and (*c*) are to guide the eye only, while in (*a*) they represent Birch–Murnaghan equations of states (Tables S17, S19 and S22). Insets in (*a*) show indicatrix plots representing compressibility tensors (with PLC and NLC marked in red and blue, respectively) calculated using *PASCal* (Cliffe & Goodwin, 2012 ▸; Lertkiattrakul *et al.*, 2023 ▸) for all data points of ETYFUM, and separately for data points of Phase I and Phase I′ of ETYSUC. The axes in the indicatrix plots show the *a*, *b* and *c* axes of the lattice.

Compression of the ETYSUC crystal up to 2.9 GPa results in a decrease in all three unit-cell parameters, and an increase in the β angle from 108.512 (3) to 113.81 (4)° (Fig. 1 ▸). Above 2.9 GPa the trend in the pressure dependence of the unit-cell parameters changes, with the *a* parameter starting to increase on compression between 2.97 and 3.73 GPa, and the slope for the parameters *c* and *b* is altered (Fig. 1 ▸). Based on this observation, it was established that above 2.9 GPa ETYSUC undergoes a phase transition to Phase I′, without alteration of the crystal symmetry nor a noticeable change in unit-cell parameters. The novel phase is structurally closely related to Phase I and differs only in its response to compression. In comparison, on compression of the ETYFUM crystal up to 3.6 GPa, only monotonic changes are observed and an increase in the length of the *a* and *c* lattice parameters and β angle accompanied by a significant decrease of *b* unit-cell parameter takes place (Fig. 1 ▸).

### 3.2. Negative linear compressibility

It was revealed that ETYSUC exhibits NLC behaviour along a similar, but quite not the same, direction (0.80**a** − 0.60**c**, Tables S17 and S18) as ETYFUM (0.73**a** − 0.68**c**, Tables S22 and S23), and of a much smaller magnitude. For the 0.1 MPa–2.9 GPa range, the median compressibility, *K*_NLC_, is equal to −5.4 (2) TPa^−1^ and the compressibility capacity (Cairns & Goodwin, 2015 ▸), χ_K_, is equal to 1.9% (Table S17, Fig. S14). Such parameters make ETYSUC a material with moderate NLC. The significantly lower magnitude is behind the observed decrease in all three unit-cell parameters, in contrast to two ETYFUM parameters that increase with compression. As the NLC strain axis is to some degree aligned with the diagonal [10−1], its elongation should affect the *a* and *c* axes in a similar manner (*i.e.* leading to an increase in the *a* and *c* unit-cell parameters). However, it can be compensated for by an increase of the β angle. The change in β angle for both cocrystals can be considered similar, and in the case of ETYSUC, where the effect of NLC is small, it is sufficient to balance the elongation along the 0.80**a** − 0.60**c** direction. Meanwhile, it is insufficient for ETYFUM. After the phase transition, when the direction of the NLC of ETYSUC slightly changes (to 0.81**a** − 0.59**c**; Table S19 and Fig. S8) and becomes aligned closer to the *a* axis, we start to observe a slight increase of the *a* parameter on compression (Fig. 1 ▸). However, for ETYSUC I′, the low number of experimental points and their inconsistency (probably caused by the crystal strain resulting from several compression/decompression runs) affect the calculated compressibility values and their estimated standard deviations (ESDs; Table S19).

As the structure of ETYSUC does not differ significantly between Phases I and I′, the strain along the principal axes was also calculated for the data of Phases I and I′ joined (Tables S20 and S21, Fig. S9). For the combined data, the NLC direction is 0.78**a** − 0.63**c**, and the median compressibility in the NLC direction shifts to −4.2 (4) TPa^−1^, which is unsurprising, as the pressure range is now wider (0.1 MPa–3.73 GPa). At the same time, compressibility values calculated for experimental points of ETYSUC I do not notably differ from the results received when ETYSUC I data were considered separately. Meanwhile, the *K*_NLC_ values obtained for Phase I′ became much more reasonable (Tables S20 and S21), supporting the observation of NLC made for Phase I′ on a limited number of data points.

#### 3.2.1. NLC mechanism

**Figure 2:**
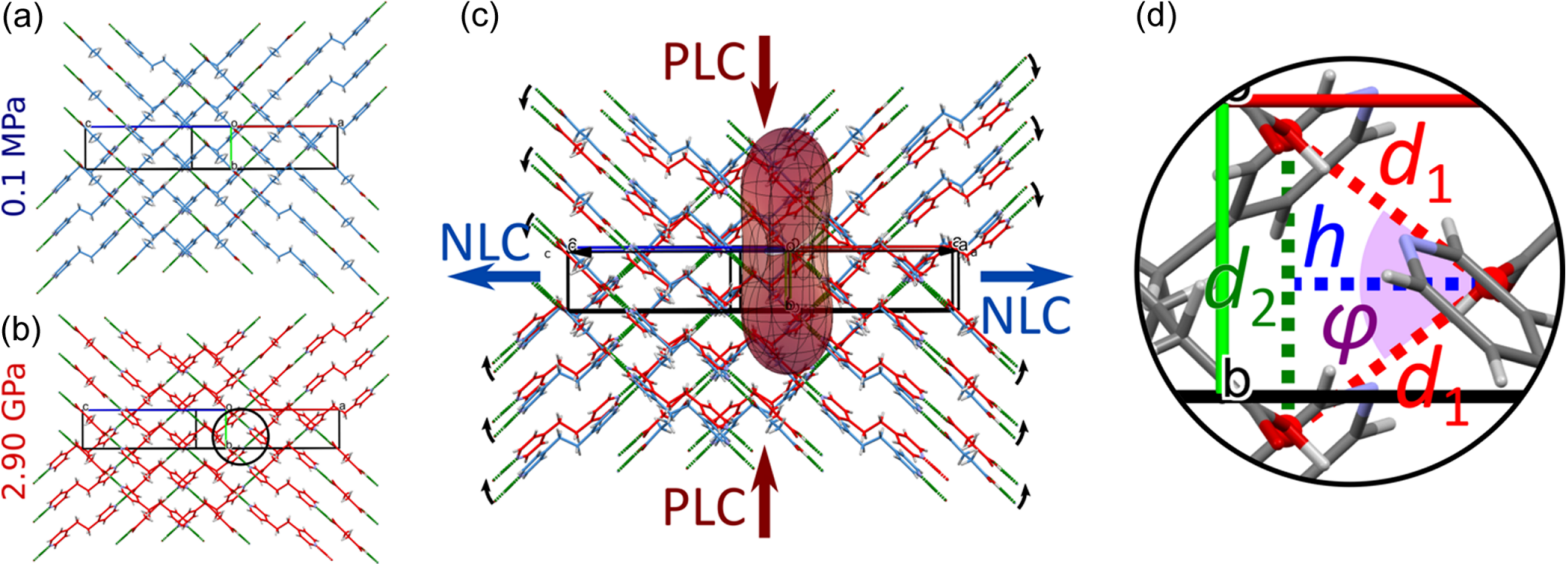
Fragment of the ETYSUC I structure at (*a*) 0.1 MPa and 298 K, and (*b*) 2.90 GPa and 298 K, showing a wine-rack motif formed by the ETY⋯SUC chains. (*c*) Superposition of the motif at 0.1 MPa (blue) and 2.90 GPa (red) with the indicatrix plot calculated using *PASCal* for the 0.1 MPa–2.90 GPa range, mapped in the same orientation as the structure (NLC marked in blue, PLC marked in red); black arrows show direction of the chains movement on compression, blue arrows the direction of NLC and red arrows the direction of PLC. (*d*) Magnification of the fragment of the ETYSUC structure showing three adjacent hinge points (red centroids) with the parameters of the triangle formed by them marked: *d*_1_ – side, red dashed lines; *d*_2_ – base, green dashed line; *h* – height, blue dashed line; φ – purple highlight.

**Figure 3:**
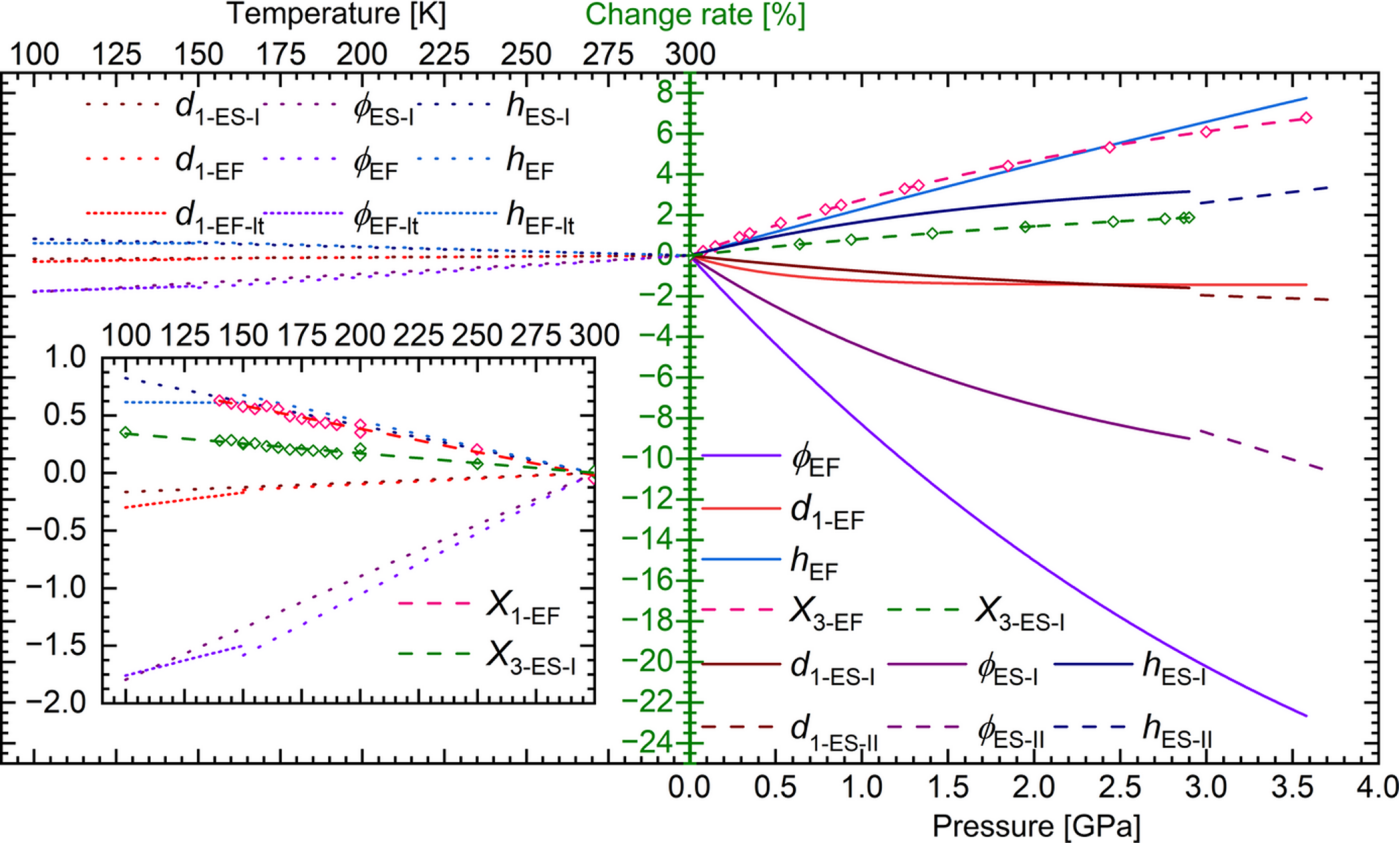
Change rate (green axis) for parameters *d*_1_, *h* and φ as a function of pressure (right) and temperature (left). For clarity, magnification of temperature data is given as an insert on the left side of the graph. In the pressure graph/magnification of the temperature graph the change in the length of the NLC axis (*X*_3_/*X*_1_) for ETYFUM and ETYSUC I, calculated using *PASCal*, is also included (pink and green open diamond symbols and dashed lines). The rate of change for the parameters *d*_1_, *h* and φ was obtained for the values calculated based on functions fitted to the experimental data (Fig. S14 and S20; Tables S33 and S35).

The mechanism behind NLC in ETYSUC is the same as in ETYFUM, *i.e.* originates from the deformation of the wine-rack motif formed by O—H⋯N bonded chains of the ETY and SUC molecules (Fig. 2 ▸). Similarly to ETYFUM, there are no classic hinges in the form of metal centres, but the same *hinge point* can be assigned: an oxygen atom of the carb­oxy­lic group interlocked between two hydrogen atoms of the pyridine ring of ETY (Figs. 2 ▸ and S10). To track the changes in the geometry of the wine rack on compression it is sufficient to analyse changes occurring in its fragment. In the case of ETYFUM, a triangle was constructed on three adjacent centroids calculated for triads of atoms O1, C4 and C5, each representing a hinge point [Figs. 2 ▸(*d*), 3 ▸ and S10]. The height of the triangle is approximately aligned with the direction of NLC in ETYFUM (0.73**a** − 0.68**c**), and is related to the parameter *d*_1_ (side) and the φ angle of the triangle according to equation (1):
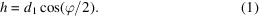
When pressure is applied, the wine rack deforms, becoming flatter, resulting in a decrease in the base of the triangle (parameter *d*_2_) and φ angle, while the side of the triangle (*d*_1_) remains almost constant (Fig. 3 ▸). Therefore, as compression progresses, the height of the triangle will increase, and as we have reported previously for ETYFUM (Patyk-Kaźmierczak & Kaźmierczak, 2024 ▸), this change matches the increase in NLC axis *X*_3_ calculated with *PASCal*, confirming the deformation of wine rack is the cause standing behind the NLC behaviour.

In the case of ETYSUC I, when we analyse the same motif as in ETYFUM, the φ angle changes by only 9% between 0.1 MPa and 2.9 GPa (compared with a decrease of more than 22% observed for ETYFUM for the 0.1 MPa–3.58 GPa range and 20% for the 0.1 MPa–2.9 GPa range). The smaller change in the φ angle translates into a weaker NLC effect. However, it should be noted that the direction of NLC in ETYSUC is different compared with ETYFUM and is not as closely aligned with the height of the triangle, which can explain the difference in the change rate of *h* and NLC axis *X*_3_ (Fig. 3 ▸). The fact that the NLC behaviour is observed and its mechanism is so strongly related to that observed in ETYFUM confirms that the latter can be used as a blueprint for NLC materials, and also shows the magnitude of the effect can be controlled.

#### 3.2.2. Managing NLC magnitude via steric hindrance

It appears that NLC damping in ETYSUC is mainly caused by a steric hindrance in the form of hydrogen atoms at the α-carbon (C2A), see Fig. S11. For ETYFUM, the pivoting of the chains can take place more freely as the hydrogen atom at the α-carbon (C8, which is an equivalent of the C2A atom in ETYSUC) is placed between the hydrogen atoms of the pyridine rings (Fig. S11). Meanwhile, atoms H5 and H6 of the pyridine ring are facing two hydrogen atoms at C2A in ETYSUC. Interestingly, when the evolution of the distance between the hydrogen atoms of the pyridine ring of ETY and the hydrogen atoms of FUM and SUC with pressure is considered (Fig. S15), we observe that it is quite similar. However, the rapprochement of the hydrogen atoms of ETY and SUC/FUM takes place not only as a result of direct compression of the crystal but also due to the pivoting of the ETY⋯SUC and ETY⋯FUM chains. Therefore, the same rapprochement of hydrogen atoms is achieved in both structures, but in ETYFUM it is associated with a decrease in the φ angle of 22% while in ETYSUC it is only by 9% (Fig. 3 ▸). It is hence clear that the distance between the hydrogen atoms of ETY and the respective acid is the limiting factor halting the pivoting of chains and hence controlling the NLC magnitude.

Interestingly, when the transition to ETYSUC I′ occurs, the strain in the form of close proximity between atoms H6 and H2ab at the symmetry-equivalent position at 1/2 + *x*, −*y*, *z* is partially released as the intermolecular distance between the two atoms increases (Fig. S12). It appears that when the distance limit between hydrogen atoms of ETY and SUC was achieved at 2.9 GPa, the structure adapted by changing the direction of NLC, allowing for further non-destructive compression of the crystal and separation of one pair of closely squeezed ETY and SUC hydrogen atoms.

**Figure 4:**
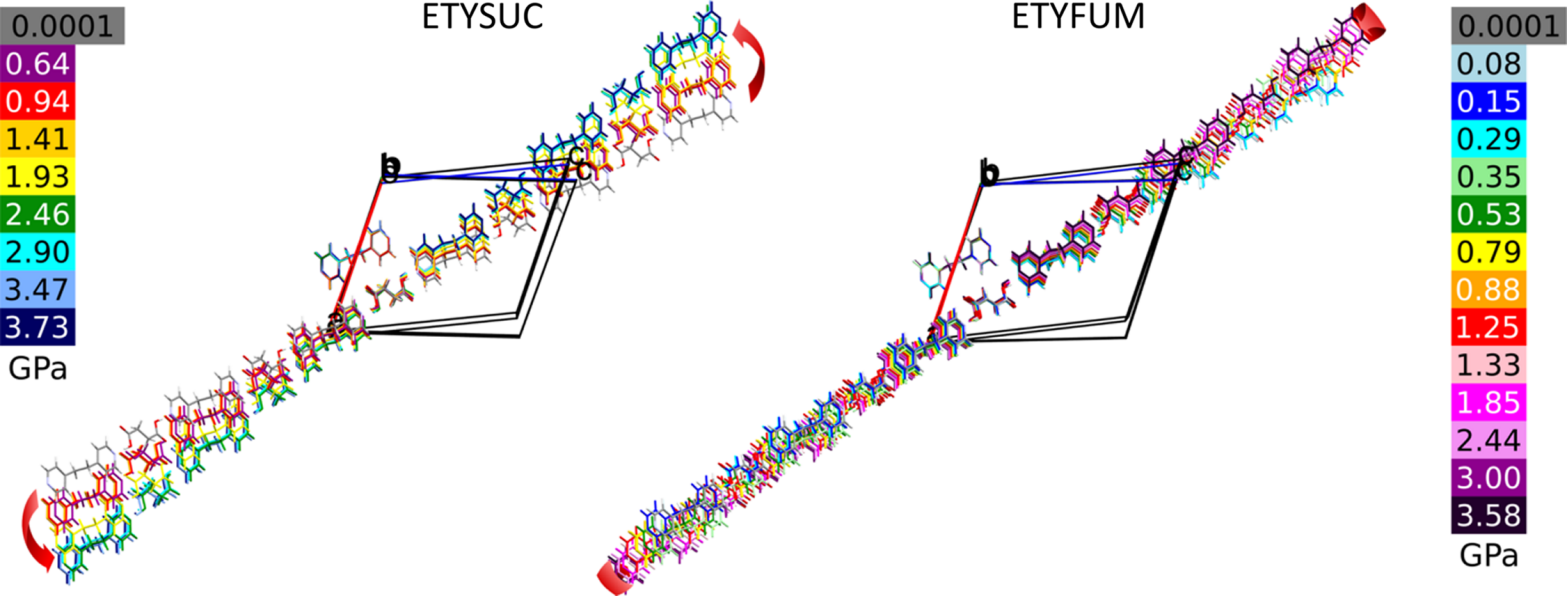
Fragments of the ETYSUC and ETYFUM structures shown along the [010] direction in the pressure ranges 0.1 MPa–3.73 GPa and 0.1 MPa–3.58 GPa, respectively. For each cocrystal, one molecule of ETY was superimposed to constitute a reference point for each structure. Structures at different pressure are marked in varied colours (see the colour scale next to each structure). Red arrows mark the direction of movement of the ETY⋯SUC/FUM chains with respect to the reference ETY molecule on compression.

Steric hindrance can also explain the difference in the direction of NLC between ETYFUM and ETYSUC. A slight rotation of the SUC molecule with respect to ETY (considering the ETY/SUC pair bonded by a C—H⋯O1 bond) helps to keep the hydrogen atoms at the α-carbon further away from the H5 and H6 atoms of ETY. As a result, the O—H⋯N bonded chains move with respect to one another in two planes instead of one, like in ETYFUM (Fig. 4 ▸). Hence, the deformation of the wine rack is accompanied by slight rotation of ETY⋯SUC chains, making the resultant NLC direction different to ETYFUM, and no longer aligned with the height of the triangle constructed on the centroids calculated for hinge points.

Lastly, some analogies between steric-hindrance control over NLC magnitude observed by us and previously reported on two hybrid perovskites {of the general formula [A]Er(HCO_2_)_2_(C_2_O_4_), where A = [(NH_2_)_3_C] or [(CH_3_)_2_NH_2_] (Hitchings *et al.*, 2024 ▸)} can be drawn. In the work by Hitchings *et. al.* (2024 ▸), only [(NH_2_)_3_C]Er(HCO_2_)_2_(C_2_O_4_) exhibits NLC, despite the wine-rack motif being present in both materials. The reasons behind this include differences in host–guest interactions, resulting from different guest molecules being present in the cavities of the framework. It appears that the presence of (CH_3_)_2_NH_2_ in the openings of the wine rack in [(CH_3_)_2_NH_2_]Er(HCO_2_)_2_(C_2_O_4_) caused steric hindrance that prevented hinging and NLC. It shows that ensuring the presence of the structural motifs predisposed to NLC might not be enough to successfully design materials of negative compressibility and additional factors such as steric hindrance need to be considered. On the other hand, it opens the possibilities to modify or avoid NLC in the wine-rack framework materials simply by exchanging guest molecules. Of course, the guest-exchange approach is not applicable to non-framework materials, such as ETYFUM or ETYSUC. In this case, the only possibility to introduce steric hindrance is to modify the wine rack by replacing molecules that form it with more bulky analogues. In the case of ETYFUM and ETYSUC, the difference in FUM and SUC molecules is extremely small, yet sufficient to significantly modify the NLC behaviour of the two materials. We believe that further exploration of the effect of larger substituents at α-carbon atoms on NLC would offer more insight into this matter. Nevertheless, our data and results reported by Hitchings *et al.* (2024 ▸) sufficiently show how the introduction of steric hindrance (either in the form of a guest molecule or structural modification of molecules forming the wine rack) can be employed to control the NLC behaviour of the material with structures utilizing the wine-rack motif, leading to either significant damping of NLC or its annihilation.

### 3.3. Negative thermal expansion

It has been previously shown that the same effects observed on crystal compression can be achieved by exposing the crystal to low temperature. However, the temperature range that can be applied is limited (with temperatures close to absolute zero being very difficult to achieve experimentally). This affects the magnitude of changes that can be induced by cooling. According to the pressure–temperature correspondence rule, usually the same effects can be achieved on compression to approximately 0.2–0.5 GPa as when the temperature is lowered from 300 to 100 K (Kaźmierczak *et al.*, 2021 ▸). Therefore, NLCs of ETYFUM and ETYSUC can be a predictor of the abnormal thermal behaviour of the two cocrystals. We have established that indeed they both exhibit NTE along one principal axis, but some differences in their behaviour can be noted.

In the case of ETYFUM, the direction of NTE (0.72**a** − 0.69**c**) is almost exactly the same as for NLC (0.73**a** − 0.68**c**); however, it is not observed over the entire investigated temperature range (100–300 K), as PTE was recorded between 100 and 150 K along all three primary axes (Tables S28–S30). Interestingly, when the crystal is gradually cooled (with 5 K steps), the NTE behaviour was observed in the 140–300 K range (Tables S28 and S29, Fig. S18). Still, there is no significant difference in the crystal structure or lattice constants of ETYFUM measured in the 140–300 K and 100–150 K ranges, and the symmetry of the crystal (the crystal system and space group) is preserved. Hence, the ETYFUM form that exists between 100 and 150 K is referenced as ETYFUM-lt (as, similarly to compressed ETYSUC, it is only the response to cooling that changes below 150 K). At the same time, NTE in ETYSUC is observed in the whole 100–300 K temperature range (Tables S24 and S25), regardless of the rate of change of temperature, and the direction of NTE in ETYSUC (0.79**a** − 0.61**c**) is also close to the direction of NLC (0.80**a** − 0.60**c**).

**Figure 5:**
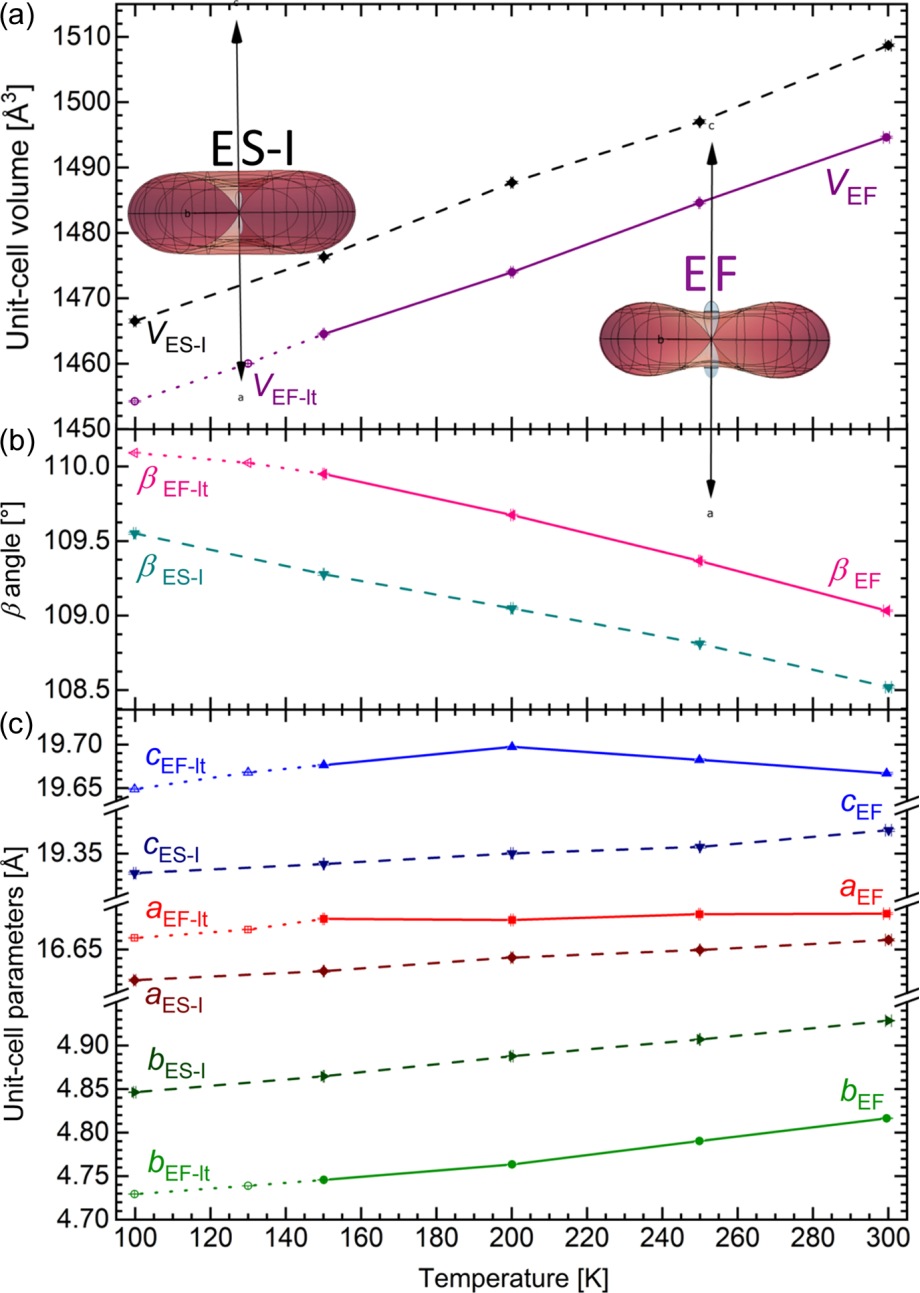
Temperature dependence of the (*a*) unit-cell volume, (*b*) β angle and (*c*) unit-cell parameters for ETYFUM sample crystal A (solid and dotted lines) and ETYSUC I sample crystal A (dashed lines). The lower subscripts EF, EF-lt and ES-I stand for ETYFUM, ETYFUM low temperature and ETYSUC Phase I, respectively. The insets in (*a*) show indicatrix plots representing thermal expansion tensors (PTE and NTE marked in red and blue, respectively) calculated using *PASCal* (Cliffe & Goodwin, 2012 ▸; Lertkiattrakul *et al.*, 2023 ▸). The axes in the indicatrix plots show the *a*, *b* and *c* axes of the lattice.

Similar to NLC, NTE is more significant in ETYFUM than in ETYSUC [with linear coefficients of thermal expansion equal to −39.7 (8) MK^−1^ and −16.5 (7) MK^−1^, respectively]. In addition, an increase in the unit-cell parameters on cooling is only observed in the case of ETYFUM, but, unlike NLC, it is recorded for the *c* unit-cell parameter only, and exclusively in the 300–200 K temperature range (Fig. 5 ▸).

#### 3.3.1. NTE mechanism and magnitude control

The mechanism behind NTE can be linked to the wine-rack motif in a manner similar to that for NLC. In general, on cooling of the ETYFUM and ETYSUC crystals, the φ angle decreases (Fig. 3 ▸, S16) while the *d*_1_ parameter remains almost constant, causing the height of the triangle to increase [according to equation (1)], which results in elongation of the crystal along one principal axis (*i.e.* NTE). Interestingly, the geometry of the wine rack changes in a similar manner on cooling ETYFUM from 150 to 100 K when the crystal exhibits PTE. However, the rate of changes of the φ angle becomes milder while the *d*_1_ parameter starts to decrease more rapidly compared with what is observed when cooling ETYFUM from 300 to 140 K and ETYSUC from 300 to 100 K. As a result, when the ETYFUM crystal is cooled from 150 to 100 K, the height of the triangle starts to decrease, which results in PTE. Moreover, in the case of ETYFUM, the increase in *h* on cooling (in the 300–140 K range) closely matches the change in the NTE axis (*X*_1_) calculated using *PASCal* (Fig. 3 ▸) which allows us to correlate NTE behaviour with deformation of the wine rack.

Similarly, as observed in the compression experiments, the height of the triangle is not aligned with the NTE axis as closely in ETYSUC as in ETYFUM. In fact, the effect of the temperature on *h* is similar for both cocrystals, but the increase of *h* in ETYSUC differs significantly from the change observed for the NTE axis *X*_3_ (see Fig. 3 ▸). Hence it appears that the final NTE magnitude might again be affected by displacement of the SUC molecules with respect to ETY, similar to that described for the compressed ETYSUC crystal. Although structural changes on cooling from 300 to 100 K are less noticeable and harder to observe visually, the temperature dependence of C—H⋯O bonds (calculated for the C—H bonds normalized to 1.089 Å to avoid bias caused by refinement of the positions of the hydrogen atoms and the lengths of the C—H bonds varying between structural models) shows that the rotation of SUC molecules with respect to ETY takes place in a manner similar to that observed for the compressed crystal of ETYSUC (Fig. S21). Although the length of both C—H⋯O bonds in ETYFUM decreases at a similar rate, in ETYSUC the length of the C5—H5⋯O1A bond decreases faster than for C6—H6⋯O1A, suggesting that the distance between atoms is affected inconsistently and is not a result of linear contraction, but rather additional rotation of molecules has to take place. As a result, the direction of NTE is affected and deformation of the triangle constructed on centroids calculated for O1, C5 and C6 atoms cannot be directly translated into the magnitude of NTE. It is therefore plausible that the effect coming from the deformation of the wine rack is damped by additional movement of SUC and ETY molecules with respect to one another.

### 3.4. Effect of pressure and temperature on torsion angles of SUC and FUM

Lastly, we would like to comment on the conformational preference of SUC molecules in ETYSUC crystals. Understandably, the order of the bond between α-carbon atoms affects the flexibility of SUC and FUM molecules. In particular, the double bond in FUM forms a conjugated system with two double carbon–oxygen bonds of carb­oxy­lic groups. As a result, there is tendency for the molecule to be planar, or nearly planar (Pauling, 1960 ▸), and it introduces a level of rigidity in the FUM molecules. In SUC the order of all carbon–carbon bonds is one and such restrictions do not apply. Nevertheless, the SUC molecules still take almost completely flat conformation [with an O1a–C1a–C2a–C2a torsion angle of about 6.2 (2)° under ambient conditions]. Despite the ability of SUC molecules to change conformation more freely, it remains resistant to changes in pressure and temperature, and on cooling to 100 K, the torsion angle oscillates in the 6.4 (4)–7.3 (5)° range (Fig. S22). The variation in torsion angle with pressure is more significant (in the 0–12° range, Fig. S22); however, as the quality of structural models is low, these values are accompanied by large ESDs and it is hard to evaluate actual changes in conformation induced by pressure. Meanwhile, the analogous torsion angle of FUM (O1–C7–C8–C8) in ETYFUM is closer to 0° [−3.2 (2)° under ambient conditions] and its response to cooling is even smaller [oscillating between −3.1 (3) and −2.7 (2)° in the 100–300 K range, see Fig. S22]. Similarly to ETYSUC, for high-pressure structural models of ETYFUM, the O1–C7–C8–C8 torsion angle of FUM shows larger variation, with values accompanied by large ESDs, which hinders reliable evaluation of the effect of pressure.

## 4. Conclusions

Abnormal pressure and temperature behaviour of the organic cocrystal ETYSUC was detected and analysed in the context of the previously reported metal-free NLC material, ETYFUM, which is isostructural to ETYSUC. Interestingly, the NLC and NTE of ETYSUC are completely concealed by the decrease in the unit-cell parameters *a*, *b* and *c* with compression and temperature reduction. Only above 2.9 GPa can an increase of the unit-cell parameter *a* be observed, and the response of the crystal to pressure changes. Hence, 2.9 GPa is considered a phase transition pressure; however, since there is no drastic change in the crystal structure, the high-pressure phase was labelled ETYSUC I′. Despite the similar molecular aggregation of ETYSUC and ETYFUM, the former exhibits NLC and NTE of significantly smaller magnitude. This can be linked to steric hindrance caused by the close positioning of hydrogen atoms at the α-carbon of SUC to hydrogen atoms H5 and H6 of the pyridine ring of ETY, and the different manner in which the O—H⋯N-bonded chains change their respective positions on compression and cooling in ETYSUC compared with ETYFUM. Despite the lower magnitude of NLC, ETYSUC can still be used as an example, confirming that ETYFUM can be used as a blueprint for the design of metal-free NLC materials. At the same time, it provides additional input to the concept by showing how steric hindrance can dampen the NLC and NTE effects and how the molecular structure needs to be considered when coformers are selected, exemplifying the macroscopic behaviour affected by microscopic modifications.

## 5. Related literature

The following references are cited in the supporting information: Budzianowski & Katrusiak (2004 ▸); Bull *et al.* (2019 ▸); Cai & Katrusiak (2014 ▸); Harty *et al.* (2015 ▸); Jiang *et al.* (2022 ▸); Knížek (2021 ▸); Macrae *et al.* (2020 ▸); Piermarini *et al.* (1975 ▸); Shephard *et al.* (2022 ▸, 2012 ▸); Szafrański (2020 ▸); Woodall *et al.* (2013 ▸); Yeung *et al.* (2017 ▸); Zhao *et al.* (2020 ▸).

## Supplementary Material

Crystal structure: contains datablock(s) etyfum_100k, etyfum_130k, etyfum_140k_b, etyfum_145k_b, etyfum_150k_b, etyfum_150k, etyfum_155k_b, etyfum_160k_b, etyfum_165k_b, etyfum_170k_b, etyfum_175k_b, etyfum_180k_b, etyfum_185k_b, etyfum_190k_b, etyfum_200k_b, etyfum_200k, ETYFUM_250K, etyfum_300k_b, etyfum_300k, ETYSUC_064, ETYSUC_094, etysuc_100K, ETYSUC_140K_B, ETYSUC_141, ETYSUC_145K_B, ETYSUC_150K_B, etysuc_150K, ETYSUC_155K_B, ETYSUC_160K_B, ETYSUC_165K_B, ETYSUC_170K_B, ETYSUC_175K_B, ETYSUC_180K_B, ETYSUC_185K_B, ETYSUC_190K_B, ETYSUC_193, ETYSUC_200K_B, etysuc_200K, ETYSUC_246, etysuc_250K, ETYSUC_276, ETYSUC_287, ETYSUC_290, ETYSUC_297, ETYSUC_300K_B, etysuc_300K, ETYSUC_329, ETYSUC_347, ETYSUC_373, etysuc. DOI: 10.1107/S2052252524011734/yc5049sup1.cif

Structure factors: contains datablock(s) etyfum_300k. DOI: 10.1107/S2052252524011734/yc5049etyfum_300ksup2.hkl

Structure factors: contains datablock(s) ETYFUM_250K. DOI: 10.1107/S2052252524011734/yc5049ETYFUM_250Ksup3.hkl

Structure factors: contains datablock(s) etyfum_200k. DOI: 10.1107/S2052252524011734/yc5049etyfum_200ksup4.hkl

Structure factors: contains datablock(s) etyfum_150k. DOI: 10.1107/S2052252524011734/yc5049etyfum_150ksup5.hkl

Structure factors: contains datablock(s) etyfum_130k. DOI: 10.1107/S2052252524011734/yc5049etyfum_130ksup6.hkl

Structure factors: contains datablock(s) etyfum_100k. DOI: 10.1107/S2052252524011734/yc5049etyfum_100ksup7.hkl

Structure factors: contains datablock(s) etyfum_300k_b. DOI: 10.1107/S2052252524011734/yc5049etyfum_300k_bsup8.hkl

Structure factors: contains datablock(s) etyfum_200k_b. DOI: 10.1107/S2052252524011734/yc5049etyfum_200k_bsup9.hkl

Structure factors: contains datablock(s) etyfum_190k_b. DOI: 10.1107/S2052252524011734/yc5049etyfum_190k_bsup10.hkl

Structure factors: contains datablock(s) etyfum_185k_b. DOI: 10.1107/S2052252524011734/yc5049etyfum_185k_bsup11.hkl

Structure factors: contains datablock(s) etyfum_180k_b. DOI: 10.1107/S2052252524011734/yc5049etyfum_180k_bsup12.hkl

Structure factors: contains datablock(s) etyfum_175k_b. DOI: 10.1107/S2052252524011734/yc5049etyfum_175k_bsup13.hkl

Structure factors: contains datablock(s) etyfum_170k_b. DOI: 10.1107/S2052252524011734/yc5049etyfum_170k_bsup14.hkl

Structure factors: contains datablock(s) etyfum_165k_b. DOI: 10.1107/S2052252524011734/yc5049etyfum_165k_bsup15.hkl

Structure factors: contains datablock(s) etyfum_160k_b. DOI: 10.1107/S2052252524011734/yc5049etyfum_160k_bsup16.hkl

Structure factors: contains datablock(s) etyfum_155k_b. DOI: 10.1107/S2052252524011734/yc5049etyfum_155k_bsup17.hkl

Structure factors: contains datablock(s) etyfum_150k_b. DOI: 10.1107/S2052252524011734/yc5049etyfum_150k_bsup18.hkl

Structure factors: contains datablock(s) etyfum_145k_b. DOI: 10.1107/S2052252524011734/yc5049etyfum_145k_bsup19.hkl

Structure factors: contains datablock(s) etyfum_140k_b. DOI: 10.1107/S2052252524011734/yc5049etyfum_140k_bsup20.hkl

Structure factors: contains datablock(s) etysuc. DOI: 10.1107/S2052252524011734/yc5049etysucsup21.hkl

Structure factors: contains datablock(s) ETYSUC_064. DOI: 10.1107/S2052252524011734/yc5049ETYSUC_064sup22.hkl

Structure factors: contains datablock(s) ETYSUC_094. DOI: 10.1107/S2052252524011734/yc5049ETYSUC_094sup23.hkl

Structure factors: contains datablock(s) ETYSUC_141. DOI: 10.1107/S2052252524011734/yc5049ETYSUC_141sup24.hkl

Structure factors: contains datablock(s) ETYSUC_193. DOI: 10.1107/S2052252524011734/yc5049ETYSUC_193sup25.hkl

Structure factors: contains datablock(s) ETYSUC_246. DOI: 10.1107/S2052252524011734/yc5049ETYSUC_246sup26.hkl

Structure factors: contains datablock(s) ETYSUC_276. DOI: 10.1107/S2052252524011734/yc5049ETYSUC_276sup27.hkl

Structure factors: contains datablock(s) ETYSUC_287. DOI: 10.1107/S2052252524011734/yc5049ETYSUC_287sup28.hkl

Structure factors: contains datablock(s) ETYSUC_290. DOI: 10.1107/S2052252524011734/yc5049ETYSUC_290sup29.hkl

Structure factors: contains datablock(s) ETYSUC_297. DOI: 10.1107/S2052252524011734/yc5049ETYSUC_297sup30.hkl

Structure factors: contains datablock(s) ETYSUC_329. DOI: 10.1107/S2052252524011734/yc5049ETYSUC_329sup31.hkl

Structure factors: contains datablock(s) ETYSUC_347. DOI: 10.1107/S2052252524011734/yc5049ETYSUC_347sup32.hkl

Structure factors: contains datablock(s) ETYSUC_373. DOI: 10.1107/S2052252524011734/yc5049ETYSUC_373sup33.hkl

Structure factors: contains datablock(s) etysuc_300K. DOI: 10.1107/S2052252524011734/yc5049etysuc_300Ksup34.hkl

Structure factors: contains datablock(s) etysuc_250K. DOI: 10.1107/S2052252524011734/yc5049etysuc_250Ksup35.hkl

Structure factors: contains datablock(s) etysuc_200K. DOI: 10.1107/S2052252524011734/yc5049etysuc_200Ksup36.hkl

Structure factors: contains datablock(s) etysuc_150K. DOI: 10.1107/S2052252524011734/yc5049etysuc_150Ksup37.hkl

Structure factors: contains datablock(s) etysuc_100K. DOI: 10.1107/S2052252524011734/yc5049etysuc_100Ksup38.hkl

Structure factors: contains datablock(s) ETYSUC_300K_B. DOI: 10.1107/S2052252524011734/yc5049ETYSUC_300K_Bsup39.hkl

Structure factors: contains datablock(s) ETYSUC_200K_B. DOI: 10.1107/S2052252524011734/yc5049ETYSUC_200K_Bsup40.hkl

Structure factors: contains datablock(s) ETYSUC_190K_B. DOI: 10.1107/S2052252524011734/yc5049ETYSUC_190K_Bsup41.hkl

Structure factors: contains datablock(s) ETYSUC_185K_B. DOI: 10.1107/S2052252524011734/yc5049ETYSUC_185K_Bsup42.hkl

Structure factors: contains datablock(s) ETYSUC_180K_B. DOI: 10.1107/S2052252524011734/yc5049ETYSUC_180K_Bsup43.hkl

Structure factors: contains datablock(s) ETYSUC_175K_B. DOI: 10.1107/S2052252524011734/yc5049ETYSUC_175K_Bsup44.hkl

Structure factors: contains datablock(s) ETYSUC_170K_B. DOI: 10.1107/S2052252524011734/yc5049ETYSUC_170K_Bsup45.hkl

Structure factors: contains datablock(s) ETYSUC_165K_B. DOI: 10.1107/S2052252524011734/yc5049ETYSUC_165K_Bsup46.hkl

Structure factors: contains datablock(s) ETYSUC_160K_B. DOI: 10.1107/S2052252524011734/yc5049ETYSUC_160K_Bsup47.hkl

Structure factors: contains datablock(s) ETYSUC_155K_B. DOI: 10.1107/S2052252524011734/yc5049ETYSUC_155K_Bsup48.hkl

Structure factors: contains datablock(s) ETYSUC_150K_B. DOI: 10.1107/S2052252524011734/yc5049ETYSUC_150K_Bsup49.hkl

Structure factors: contains datablock(s) ETYSUC_145K_B. DOI: 10.1107/S2052252524011734/yc5049ETYSUC_145K_Bsup50.hkl

Structure factors: contains datablock(s) ETYSUC_140K_B. DOI: 10.1107/S2052252524011734/yc5049ETYSUC_140K_Bsup51.hkl

Supporting data, figures and tables. DOI: 10.1107/S2052252524011734/yc5049sup52.pdf

CCDC references: 2411299, 2411300, 2411301, 2411302, 2411303, 2411304, 2411305, 2411306, 2411307, 2411308, 2411309, 2411310, 2411311, 2411312, 2411313, 2411314, 2411315, 2411316, 2411317, 2411318, 2411319, 2411320, 2411321, 2411322, 2411323, 2411324, 2411325, 2411326, 2411327, 2411328, 2411329, 2411330, 2411331, 2411332, 2411333, 2411334, 2411335, 2411336, 2411337, 2411338, 2411339, 2411340, 2411341, 2411342, 2411343, 2411344, 2411345, 2411346, 2411347, 2411348
