# Supplementary material for: Managing negative linear compressibility and thermal expansion through steric hindrance: a case study of 1,2-bis­(4′-pyridyl)­ethane cocrystals
Source: IUCrJ. 2025 Jan 1;12(Pt 1):88–96. doi: 10.1107/S2052252524011734 (PMC11707692; doi:10.1107/S2052252524011734)
Supplement: Supplementary file 52 [file m-12-00088-sup52.pdf]

IUCrJ

Volume 12 (2025)

Supporting information for article:

Managing negative linear compressibility and thermal expansion through steric hindrance: a case study of 1,2-bis(4′-pyridyl)ethane cocrystals

Ewa Patyk-Kaźmierczak, Kornelia Szymańska and Michał Kaźmierczak

S1. Experimental details

S1.1. Cocrystal synthesis

S1.1.1. Slow evaporation

ETYSUC: 18 mg of 1,2-bis(4'-pyridyl)ethane (ETY) and 11 mg of succinic acid (SUC) (approx. 1:1 mol. ratio) were dissolved in 5 ml of hot methanol (heated slightly above boiling point) and then left for slow evaporation at room temperature in fuming hood. The good quality crystals emerged after couple of days.

ETYFUM: 18.4 mg of 1,2-bis(4'-pyridyl)ethane (ETY) and 11.6 mg of fumaric acid (FUM) (approx. 1:1 mol. ratio) were dissolved in hot methanol (heated slightly above boiling point) and left for slow evaporation at room temperature in fuming hood. The high quality single crystals emerged after couple of days.

S1.1.2. Solvent-assisted ball milling

61 mg of ETY and 39 mg of SUC (1:1 mol. ratio) were mixed and placed in a 6 ml in-house made Teflon milling cup alongside Teflon ball (5 mm in diameter) and 10 µl of 99.8% methanol (analytically pure). Sample was then milled for 30 minutes (at 30 Hz frequency) at room temperature using 400 MM ball mill from Retsch. After completion of the milling sample was measured using powder X-ray diffraction.

S1.2. X-ray diffraction experiments

S1.2.1. Single crystal X-ray diffraction (SCXRD)

Ambient conditions: A single crystal of ETYSUC (0.2x0.1x0.04 mm) suitable for SCXRD experiment was mounted on a MiTeGen loop and measured with SuperNova four-circle single crystal diffractometer from Rigaku Oxford Diffraction, equipped in mirror monochromator, micro-focus sealed X-ray tube (Cu K_{α}=1.54184 Å) and Atlas detector. Data were collected using CrysAlisPro (Rigaku Oxford Diffraction, 2020) program at 298 K.

Low-temperature: Two sample crystals suitable for SCXRD experiments were selected for ETYSUC and ETYFUM each (Figures S2-S5). Each sample crystal was mounted on a MiTeGen loop and measured either with SuperNova four-circle single crystal diffractometer from Rigaku Oxford Diffraction (equipped in mirror monochromator, copper micro-focus sealed X-ray tube of K_{α}=1.54184 Å, Atlas detector) – experimental series A, or with New Excalibur four-cicle diffractometer from Rigaku Oxford Diffraction (equipped in graphite monochromator, copper fine-focus sealed X-ray tube of K_{α}=1.54184 Å, EosS2 detector). All data were collected using CrysAlisPro (Rigaku Oxford Diffraction, 2020) program. Low temperature conditions were achieved using the nitrogen-flow Oxford

Cryosystem attachments mounted at each diffractometer. For the ETYFUM sample crystal A data were collected at 300, 250, 200, 150, 130 and 100 K and for sample crystal B at 300, 200, 190, 185, 180, 175, 170, 165, 160, 155, 150, 145 and 140 K. For ETYSUC sample crystal A data were collected at 300, 250, 200, 150 and 100 K and for sample crystal B at 300, 200, 190, 185, 180, 175, 170, 165, 160, 155, 150, 145 and 140 K.

High-pressure: An ETYSUC single crystal was loaded in an opening (0.41 mm in diameter) of a preindented steel gasket (0.3 mm thick) mounted in modified Merrill-Bassett (Merrill & Bassett, 1974) diamond anvil cell (DAC). Alongside sample crystal, a ruby chip (used for pressure measurement) and cellulose fibres (used to prevent crystal movement during the experiment) were placed in the opening. So prepared chamber was filled with Daphne 7575 oil (ensuring hydrostatic conditions up to c.a. 4 GPa(Daphne Oil 7575 Data sheet)), used as pressure-transmitting medium (PTM) and then DAC was tightly closed. The sample underwent gradual compression (interrupted by a decompression step) with X-ray diffraction experiment performed for sample under following pressure conditions (listed in the chronological order of the measurements): 0.64(2), 0.94(2), 1.41(2), 1.93(2), 2.46(2), 2.97(2), 3.47(2), 3.73(2), 2.76(2), 2.87(2), 2.90(2), 3.29(2) GPa (Figure S1).

BETSA PRL spectrometer equipped in Photon Control Inc. detector (affording accuracy of approx. 0.02 GPa) was used to measure the pressure inside the DAC (based on the shift of ruby fluorescence line (Piermarini *et al.*, 1975)). A four-circle single crystal Xcalibur X-ray diffractometer from Rigaku Oxford diffraction (equipped in graphite monochromator, molybdenum fine-focus sealed X-ray tube of K_{α}=0.71073 Å, EOS CCD detector) was used for all data collection. Gasket shadowing method (Budzianowski & Katrusiak, 2004) was applied to centre the DAC prior to X-ray measurement, and all data was collected with program CrysAlisPro (Rigaku Oxford Diffraction, 2020).

S1.2.2. Powder X-ray diffraction (PXRD)

A powder sample (from solvent-assisted ball milling synthesis) was measured using Bruker D8 Advanced diffractometer (equipped in copper X-ray tube of $K_{\alpha 1}$=1.5406 Å, Johnsson monochromator and LynxEye strip detector). The scan was performed for 5-50° 2θ angle range. Program Kdif (from Kalvados software package (Knížek, 2021)) was used for data processing. The experimental PXRD pattern was plotted using OriginPro 2022b (Figure S6) alongside PXRD pattern calculated based on crystal structure of ETYSUC at 298 K/0.1 MPa. The simulated pattern for ETYSUC was produced with program Mercury (Macrae *et al.*, 2020).

S1.3. Crystal structure solution

For all data, program CrysAlisPro (Rigaku Oxford Diffraction, 2020) was used to determine *UB*-matrix, reduce data and to apply absorption corrections. Crystal structures were solved by intrinsic phasing (with ShelXT; Sheldrick, 2015*b*)) or by direct methods (with SHELXS; Sheldrick, 2008)) and refined

with least-squares method (with ShelXL; Sheldrick, 2015*a*)), with Olex2 (Dolomanov *et al.*, 2009) used as an interface. The structural models for ambient and low-temperature data were refined using anisotropic thermal factors, and the positions of hydrogen atoms were assigned and refined based on electron density. In case of high-pressure structures, the hydrogen atoms were located at idealized positions, based on the molecular geometry, with isotropic thermal parameters depending on the equivalent displacement parameters of their carriers. The low number of collected data caused by the restricted access of the incident X-ray beam to the sample (unfavourably affecting data/parameter ratio) hindered anisotropic refinement of structural models for high-pressure data. Therefore high-pressure structures were only refined isotropically. All crystal structures were deposited with the Cambridge Crystallographic Data Centre (CCDC: 2383677-2383682, 2383684-2383709, 2383846-2383863) and can be accessed free of charge by filling out an online form at https://www.ccdc.cam.ac.uk/structures/, or can be found in Zenodo repository (DOI: 10.5281/zenodo.14382410). Crystallographic details for all structures are listed in Tables S1-S16.

S1.4. Principal axis strain and linear coefficient of thermal expansion calculations

Program PASCal (Cliffe & Goodwin, 2012; Lertkiattrakul *et al.*, 2023), available at https://www.pascalapp.co.uk/, was used to calculate principal axis strain for ETYSUC I, ETYSUC I', ETYSUC all data (phase I and I' combined), and to calculate linear coefficients of thermal expansion for ETYSUC I and ETYFUM. Principal axis strain for ETYFUM is cited after Patyk-Kaźmierczak & Kaźmierczak (2024). The ESDs on pressure values were used as weights for each data point. Details on how weights were applied in each case are listed in the comment's section of each table summarizing calculations results (Tables S18-S20). An indicatrix plots showing principal axis strain and thermal expansion are shown in Figures S7-S11.

In all cases of principal axis strain calculations, a finite Eulerian strain was calculated and an empirical equation-of-state $\epsilon(p) = \epsilon_0 + \lambda(p - p_c)^{\nu}$ was fitted to the strain eigenvalues. The compressibility vs. pressure plots for all ETYSUC I, ETYSUC all data and ETYFUM are shown in graph in Figure S12.

S1.5. Compressibility capacity calculations

Compressibility capacity (χ_K) for ETYSUC I, and phases I and I' combined was calculated using functions fitted by PASCal to negative strain eigenvalues. Those functions were exported and plotted in function of pressure, and then integrated for the total investigated pressure range (0.1 MPa- 2.90 GPa for ETYSUC I, and 0.1 MPa-3.73 GPa for ETYSUC I and I' combined) using program OriginPro(OriginPro). The results are shown graphically in Figure S13. To obtain the final values of compressibility capacity (χ_K, defined as $\chi_K = -\int_{p_{\min}}^{p_{\max}} K(p)\mathrm{d}p$), and expressed as a percentage [%], the area values obtained after integration were multiplied by -1, and by 10^{-1} to account for the

discrepancy in pressure units (in the plots compressibility is expressed in TPa^{-1} and pressure is expressed in GPa). The calculated values of compressibility capacity for ETYSUC are listed in Table S31, alongside previously reported χ_K values for selected NLC materials.

S2. Tables

S2.1. Experimental details

Table S1 Experimental and crystallographic details for ETYSUC I at ambient conditions.

	ETYSUC
Crystal data	
Chemical formula	$C_{12}H_{12}N_2 \cdot C_4H_6O_4$
M_r	302.32
Crystal system, space group	Monoclinic, *I*2/*a*
Temperature (K)	295
a, *b*, *c* (Å)	16.6711 (3), 4.9272 (1), 19.3744 (4)
β (°)	108.512 (2)
V (Å^3)	1509.10 (5)
Z	4
Radiation type	Cu *K*α
μ (mm^{-1})	0.80
Crystal size (mm)	0.2 × 0.1 × 0.04
Data collection	
Diffractometer	SuperNova, Single source at offset/far, Atlas
Absorption correction	Gaussian *CrysAlis PRO* 1.171.42.49 (Rigaku Oxford Diffraction, 2022) Numerical absorption correction based on gaussian integration over a multifaceted crystal model Empirical absorption correction using spherical harmonics, implemented in SCALE3 ABSPACK scaling algorithm.
T_{min}, T_{max}	0.813, 1.000
No. of measured, independent and observed [$I > 2\sigma(I)$] reflections	12238, 1554, 1318
R_{int}	0.027
$(\sin \theta/\lambda)_{max}$ (Å^{-1})	0.630
Refinement	
$R[F^2 > 2\sigma(F^2)]$, $wR(F^2)$, S	0.037, 0.105, 1.03
No. of reflections	1554
No. of parameters	137
H-atom treatment	All H-atom parameters refined
$\Delta\rho_{max}$, $\Delta\rho_{min}$ (e Å^{-3})	0.17, −0.16

Computer programs: *CrysAlis PRO* 1.171.42.49 (Rigaku OD, 2022), SHELXT 2014/4 (Sheldrick, 2014), *SHELXL* 2014/7 (Sheldrick, 2015), Olex2 1.5 (Dolomanov *et al.*, 2009).

Table S2 Experimental and crystallographic details for ETYSUC I (high-pressure experiments 0.64-1.93 GPa pressure range). For all structures: $C_{12}H_{12}N_2{\cdot}C_4H_6O_4$, M_r = 302.32, monoclinic, *I*2/*a*, *Z* = 4. Experiments were carried out at 295 K with Mo *K*α radiation using a Xcalibur, Eos. Gaussian, *CrysAlis PRO* 1.171.42.49 (Rigaku Oxford Diffraction, 2022) Absorption was corrected for by numerical methods absorption correction based on gaussian integration over a multifaceted crystal model (Absorb Angel (2004) J. Appl. Cryst. 37:486-492). Refinement was on 46 parameters. H-atom parameters were constrained.

	ETYSUC_064	ETYSUC_094	ETYSUC_141	ETYSUC_193
Crystal data				
Pressure (kPa)	640000	940000	1410000	1930000
a, *b*, *c* (Å)	16.60 (4), 4.7781 (4), 19.218 (9)	16.56 (3), 4.7226 (3), 19.187 (7)	16.55 (3), 4.6266 (3), 19.089 (6)	16.53 (2), 4.5612 (3), 19.065 (7)
β (°)	110.32 (13)	110.99 (11)	111.96 (9)	112.63 (10)
V (Å^3)	1429 (4)	1401 (3)	1356 (2)	1327 (2)
Radiation type	Mo *K*α	Mo *K*α	Mo *K*α	Mo *K*α
μ (mm^{-1})	0.10	0.10	0.11	0.11
Crystal size (mm)	0.35 × 0.16 × 0.03	0.35 × 0.16 × 0.03	0.37 × 0.16 × 0.03	0.35 × 0.16 × 0.03
Data collection				
T_{min}, T_{max}	0.996, 0.997	0.996, 0.997	0.996, 0.996	0.996, 0.996
No. of measured, independent and observed [$I > 2\sigma(I)$] reflections	1787, 283, 167	2114, 307, 165	1943, 282, 177	1144, 292, 159
R_{int}	0.089	0.099	0.099	0.087
$(\sin \theta/\lambda)_{max}$ (Å^{-1})	0.613	0.621	0.622	0.619
Refinement				
$R[F^2 > 2\sigma(F^2)]$, $wR(F^2)$, S	0.080, 0.227, 1.07	0.087, 0.265, 1.12	0.091, 0.274, 1.10	0.089, 0.291, 1.07
No. of reflections	283	307	282	292
No. of restraints	5	6	9	8
$\Delta\rho_{max}$, $\Delta\rho_{min}$ (e Å^{-3})	0.17, −0.21	0.19, −0.28	0.22, −0.29	0.26, −0.27

Computer programs: *CrysAlis PRO* 1.171.42.49 (Rigaku OD, 2022), SHELXT 2014/4 (Sheldrick, 2014), olex2.solve 1.5 (Bourhis *et al.*, 2015), *SHELXS* (Sheldrick, 2008), *SHELXL* 2014/7 (Sheldrick, 2015), Olex2 1.5 (Dolomanov *et al.*, 2009).

Table S3 Experimental and crystallographic details for ETYSUC I (high-pressure experiments 2.46-1-2.90 GPa pressure range). For all structures: C12H12N2·C4H6O4, Mr = 302.32, monoclinic, I2/a, Z = 4. Experiments were carried out at 295 K with Mo Kα radiation using a Xcalibur, Eos. Gaussian, CrysAlis PRO 1.171.42.49 (Rigaku Oxford Diffraction, 2022) Absorption was corrected for by numerical methods absorption correction based on gaussian integration over a multifaceted crystal model (Absorb Angel (2004) J. Appl. Cryst. 37:486-492). Refinement was on 46 parameters. H-atom parameters were constrained.

	ETYSUC_246	ETYSUC_276	ETYSUC_287	ETYSUC_290
Crystal data				
Pressure (kPa)	2460000	2760000	2870000	2900000
a, *b*, *c* (Å)	16.48 (4), 4.5205 (4), 19.083 (12)	16.469 (14), 4.4857 (2), 19.042 (4)	16.462 (12), 4.4828 (2), 19.038 (3)	16.446 (10), 4.46879 (18), 19.038 (3)
β (°)	113.24 (16)	113.63 (5)	113.66 (5)	113.81 (4)
V (Å^3)	1307 (4)	1288.7 (12)	1286.9 (10)	1280.2 (9)
μ (mm^{-1})	0.11	0.11	0.11	0.11
Crystal size (mm)	0.35 × 0.16 × 0.03	0.31 × 0.15 × 0.03	0.31 × 0.15 × 0.03	0.31 × 0.15 × 0.03
Data collection				
T_{min}, T_{max}	0.996, 0.996	0.995, 0.996	0.995, 0.996	0.995, 0.996
No. of measured, independent and observed [$I > 2\sigma(I)$] reflections	1924, 259, 163	4834, 361, 229	3855, 366, 215	2572, 314, 211
R_{int}	0.076	0.081	0.086	0.076
$(\sin \theta/\lambda)_{max}$ (Å^{-1})	0.624	0.634	0.630	0.631
Refinement				
$R[F^2 > 2\sigma(F^2)]$, $wR(F^2)$, S	0.058, 0.187, 1.11	0.088, 0.281, 1.13	0.086, 0.270, 1.02	0.060, 0.174, 1.05
No. of reflections	259	361	366	314
No. of restraints	9	5	5	5
$\Delta\rho_{max}$, $\Delta\rho_{min}$ (e Å^{-3})	0.14, −0.17	0.22, −0.30	0.19, −0.25	0.15, −0.21

Computer programs: CrysAlis PRO 1.171.42.49 (Rigaku OD, 2022), olex2.solve 1.5 (Bourhis et al., 2015), SHELXS (Sheldrick, 2008), SHELXL 2014/7 (Sheldrick, 2015), Olex2 1.5 (Dolomanov et al., 2009)

Table S4 Experimental and crystallographic details for ETYSUC I' (high pressure experiments). For all structures: C12H12N2·C4H6O4, Mr = 302.32, monoclinic, I2/a, Z = 4. Experiments were carried out at 295 K with Mo Kα radiation. Gaussian, CrysAlis PRO 1.171.42.49 (Rigaku Oxford Diffraction, 2022) Absorption was corrected for by numerical methods absorption correction based on gaussian integration over a multifaceted crystal model (Absorb Angel (2004) J. Appl. Cryst. 37:486-492). Refinement was on 46 parameters with 4 restraints. H-atom parameters were constrained.

	ETYSUC_297	ETYSUC_329	ETYSUC_347	ETYSUC_373
Crystal data				
Pressure (kPa)	2970000	3290000	3470000	3730000
a, *b*, *c* (Å)	16.33 (7), 4.4824 (9), 19.06 (2)	16.429 (12), 4.4214 (3), 19.020 (4)	16.44 (9), 4.4104 (11), 18.97 (3)	16.46 (6), 4.3839 (7), 18.94 (2)
β (°)	113.4 (3)	114.32 (5)	114.0 (4)	114.3 (3)
V (Å^3)	1280 (7)	1258.9 (11)	1256 (8)	1246 (6)
μ (mm^{-1})	0.11	0.12	0.12	0.12
Crystal size (mm)	0.19 × 0.09 × 0.02	0.31 × 0.15 × 0.03	0.19 × 0.09 × 0.02	0.19 × 0.09 × 0.02
Data collection				
Diffractometer	New Xcalibur, EosS2	Xcalibur, Eos	New Xcalibur, EosS2	New Xcalibur, EosS2
T_{min}, T_{max}	0.997, 0.998	0.995, 0.997	0.998, 0.998	0.997, 0.998
No. of measured, independent and observed [$I > 2\sigma(I)$] reflections	1082, 252, 164	4135, 353, 241	1431, 289, 181	1000, 253, 159
R_{int}	0.097	0.082	0.095	0.095
(sin θ/λ)$_{max}$ (Å^{-1})	0.614	0.635	0.623	0.622
Refinement				
$R[F^2 > 2\sigma(F^2)]$, $wR(F^2)$, S	0.073, 0.227, 1.04	0.062, 0.162, 1.07	0.066, 0.187, 1.11	0.090, 0.271, 1.04
No. of reflections	252	353	289	253
$\Delta\rho_{max}$, $\Delta\rho_{min}$ (e Å^{-3})	0.23, −0.28	0.18, −0.23	0.16, −0.32	0.24, −0.34

Computer programs: *CrysAlis PRO* 1.171.42.49 (Rigaku OD, 2022), *SHELXL* 2014/7 (Sheldrick, 2015), Olex2 1.5 (Dolomanov *et al.*, 2009).

Table S5 Experimental and crystallographic details for ETYSUC I sample crystal A (in 300-150 K temperature range). For all structures: C12H12N2·C4H6O4, Mr = 302.32, monoclinic, I2/a, Z = 4. Experiments were carried out with Cu Kα radiation using a SuperNova, Single source at offset/far, Atlas. Gaussian, CrysAlis PRO 1.171.42.49 (Rigaku Oxford Diffraction, 2022) Absorption was corrected for by numerical methods absorption correction based on gaussian integration over a multifaceted crystal model Empirical absorption correction using spherical harmonics, implemented in SCALE3 ABSPACK scaling algorithm. All H-atom parameters were refined.

	etysuc_300K	etysuc_250K	etysuc_200K	etysuc_150K
Crystal data				
Temperature (K)	300	250	200	150
a, b, c (Å)	16.6610 (13), 4.9284 (4), 19.3767 (13)	16.6494 (14), 4.9069 (4), 19.3575 (13)	16.6408 (10), 4.8878 (3), 19.3501 (10)	16.6252 (13), 4.8647 (4), 19.3379 (11)
β (°)	108.520 (8)	108.812 (9)	109.048 (6)	109.278 (8)
V (Å^3)	1508.7 (2)	1497.0 (2)	1487.70 (16)	1476.3 (2)
μ (mm^{-1})	0.80	0.81	0.81	0.82
Crystal size (mm)	0.14 × 0.09 × 0.04	0.14 × 0.09 × 0.04	0.14 × 0.09 × 0.04	0.14 × 0.09 × 0.04
Data collection				
T_{min}, T_{max}	0.907, 1.000	0.904, 1.000	0.903, 1.000	0.903, 1.000
No. of measured, independent and observed [$I > 2\sigma(I)$] reflections	2850, 1444, 914	2831, 1432, 961	2799, 1422, 953	2791, 1416, 954
R_{int}	0.034	0.036	0.041	0.037
$(\sin \theta/\lambda)_{max}$ (Å^{-1})	0.618	0.618	0.618	0.617
Refinement				
$R[F^2 > 2\sigma(F^2)]$, $wR(F^2)$, S	0.052, 0.154, 1.01	0.053, 0.165, 1.02	0.059, 0.188, 1.09	0.060, 0.190, 1.04
No. of reflections	1444	1432	1422	1416
No. of parameters	136	136	136	137
$\Delta\rho_{max}$, $\Delta\rho_{min}$ (e Å^{-3})	0.15, −0.19	0.17, −0.18	0.19, −0.29	0.25, −0.25

Computer programs: *CrysAlis PRO* 1.171.42.49 (Rigaku OD, 2022), SHELXT 2014/4 (Sheldrick, 2014), *SHELXL* 2014/7 (Sheldrick, 2015), Olex2 1.5 (Dolomanov *et al.*, 2009).

Table S6 Experimental and crystallographic details for ETYSUC I sample crystal A (at 100 K). For all structures: C12H12N2·C4H6O4, Mr = 302.32, monoclinic, I2/a, Z = 4. Experiments were carried out with Cu Kα radiation using a SuperNova, Single source at offset/far, Atlas. Gaussian, CrysAlis PRO 1.171.42.49 (Rigaku Oxford Diffraction, 2022) Absorption was corrected for by numerical methods absorption correction based on gaussian integration over a multifaceted crystal model Empirical absorption correction using spherical harmonics, implemented in SCALE3 ABSPACK scaling algorithm. All H-atom parameters were refined.

	etysuc_100K
Crystal data	
Temperature (K)	100
a, *b*, *c* (Å)	16.6147 (11), 4.8463 (4), 19.3274 (11)
β (°)	109.551 (7)
V (Å^3)	1466.51 (19)
μ (mm^{-1})	0.82
Crystal size (mm)	0.14 × 0.09 × 0.04
Data collection	
T_{min}, T_{max}	0.898, 1.000
No. of measured, independent and observed [$I > 2\sigma(I)$] reflections	2776, 1405, 997
R_{int}	0.054
$(\sin \theta/\lambda)_{max}$ (Å^{-1})	0.617
Refinement	
$R[F^2 > 2\sigma(F^2)]$, $wR(F^2)$, S	0.065, 0.187, 1.03
No. of reflections	1405
No. of parameters	137
$\Delta\rho_{max}$, $\Delta\rho_{min}$ (e Å^{-3})	0.35, −0.31

Computer programs: *CrysAlis PRO* 1.171.42.49 (Rigaku OD, 2022), SHELXT 2014/4 (Sheldrick, 2014), *SHELXL* 2014/7 (Sheldrick, 2015), Olex2 1.5 (Dolomanov *et al.*, 2009).

Table S7 Experimental and crystallographic details for ETYSUC I sample crystal B (in 300-185 K temperature range). For all structures: $C_{12}H_{12}N_2{\cdot}C_4H_6O_4$, M_r = 302.32, monoclinic, *I*2/*a*, *Z* = 4. Experiments were carried out with Cu *K*α radiation using a New Xcalibur, EosS2. Gaussian, *CrysAlis PRO* 1.171.42.49 (Rigaku Oxford Diffraction, 2022) Absorption was corrected for by numerical methods absorption correction based on gaussian integration over a multifaceted crystal model Empirical absorption correction using spherical harmonics, implemented in SCALE3 ABSPACK scaling algorithm. Refinement was on 136 parameters. All H-atom parameters were refined.

	ETYSUC_300K_B	ETYSUC_200K_B	ETYSUC_190K_B	ETYSUC_185K_B
Crystal data				
Temperature (K)	300	200	190	185
a, *b*, *c* (Å)	16.6688 (5), 4.9269 (1), 19.3733 (6)	16.6313 (5), 4.8871 (1), 19.3441 (6)	16.6279 (3), 4.8825 (1), 19.3411 (4)	16.6268 (3), 4.8810 (1), 19.3397 (4)
β (°)	108.522 (3)	109.050 (3)	109.105 (2)	109.141 (2)
V ($Å^3$)	1508.63 (8)	1486.16 (8)	1483.73 (5)	1482.75 (5)
μ (mm^{-1})	0.80	0.81	0.81	0.81
Crystal size (mm)	0.47 × 0.41 × 0.12	0.47 × 0.41 × 0.12	0.44 × 0.42 × 0.15	0.47 × 0.41 × 0.12
Data collection				
T_{min}, T_{max}	0.767, 0.923	0.765, 0.923	0.764, 0.904	0.764, 0.922
No. of measured, independent and observed [$I > 2\sigma(I)$] reflections	4278, 1432, 1276	4155, 1415, 1283	4224, 1415, 1295	4221, 1414, 1300
R_{int}	0.023	0.022	0.024	0.025
$(\sin \theta/\lambda)_{max}$ ($Å^{-1}$)	0.615	0.615	0.615	0.615
Refinement				
$R[F^2 > 2\sigma(F^2)]$, $wR(F^2)$, S	0.048, 0.142, 1.07	0.044, 0.125, 1.06	0.044, 0.124, 1.07	0.043, 0.121, 1.05
No. of reflections	1432	1415	1415	1414
$\Delta\rho_{max}$, $\Delta\rho_{min}$ (e $Å^{-3}$)	0.18, −0.32	0.19, −0.27	0.18, −0.29	0.19, −0.29

Computer programs: *CrysAlis PRO* 1.171.42.49 (Rigaku OD, 2022), SHELXT 2014/4 (Sheldrick, 2014), *SHELXL* 2019/3 (Sheldrick, 2015), Olex2 1.5 (Dolomanov *et al.*, 2009).

Table S8 Experimental and crystallographic details for ETYSUC I sample crystal B (in 180-170 K temperature range). For all structures: $C_{12}H_{12}N_2{\cdot}C_4H_6O_4$, M_r = 302.32, monoclinic, *I*2/*a*, *Z* = 4. Experiments were carried out with Cu *K*α radiation using a New Xcalibur, EosS2. Gaussian, *CrysAlis PRO* 1.171.42.49 (Rigaku Oxford Diffraction, 2022) Absorption was corrected for by numerical methods absorption correction based on gaussian integration over a multifaceted crystal model Empirical absorption correction using spherical harmonics, implemented in SCALE3 ABSPACK scaling algorithm. Refinement was on 136 parameters. All H-atom parameters were refined.

	ETYSUC_180K_B	ETYSUC_175K_B	ETYSUC_170K_B
Crystal data			
Temperature (K)	180	175	170
a, *b*, *c* (Å)	16.6256 (3), 4.8792 (1), 19.3393 (4)	16.6216 (4), 4.8770 (1), 19.3388 (4)	16.6197 (3), 4.8747 (1), 19.3362 (4)
β (°)	109.164 (2)	109.193 (2)	109.217 (2)
V ($Å^3$)	1481.86 (5)	1480.53 (6)	1479.25 (5)
μ (mm^{-1})	0.81	0.81	0.82
Crystal size (mm)	0.47 × 0.41 × 0.12	0.47 × 0.41 × 0.12	0.47 × 0.41 × 0.12
Data collection			
T_{min}, T_{max}	0.764, 0.922	0.764, 0.922	0.764, 0.922
No. of measured, independent and observed [$I > 2\sigma(I)$] reflections	4217, 1413, 1286	4215, 1411, 1288	4216, 1410, 1291
R_{int}	0.023	0.023	0.023
$(\sin \theta/\lambda)_{max}$ ($Å^{-1}$)	0.614	0.615	0.615
Refinement			
$R[F^2 > 2\sigma(F^2)]$, $wR(F^2)$, S	0.044, 0.123, 1.06	0.043, 0.122, 1.06	0.042, 0.120, 1.04
No. of reflections	1413	1411	1410
$\Delta\rho_{max}$, $\Delta\rho_{min}$ (e $Å^{-3}$)	0.19, −0.30	0.21, −0.29	0.19, −0.28

Computer programs: *CrysAlis PRO* 1.171.42.49 (Rigaku OD, 2022), SHELXT 2014/4 (Sheldrick, 2014), *SHELXL* 2019/3 (Sheldrick, 2015), Olex2 1.5 (Dolomanov *et al.*, 2009).

Table S9 Experimental and crystallographic details for ETYSUC I sample crystal B (in 165-150 K temperature range). For all structures: $C_{12}H_{12}N_2{\cdot}C_4H_6O_4$, M_r = 302.32, monoclinic, *I*2/*a*, *Z* = 4. Experiments were carried out with Cu *K*α radiation using a New Xcalibur, EosS2. Gaussian, *CrysAlis PRO* 1.171.42.49 (Rigaku Oxford Diffraction, 2022) Absorption was corrected for by numerical methods absorption correction based on gaussian integration over a multifaceted crystal model Empirical absorption correction using spherical harmonics, implemented in SCALE3 ABSPACK scaling algorithm. Refinement was on 136 parameters. All H-atom parameters were refined.

	ETYSUC_165K_B	ETYSUC_160K_B	ETYSUC_155K_B	ETYSUC_150K_B
Crystal data				
Temperature (K)	165	160	155	150
a, *b*, *c* (Å)	16.6187 (3), 4.8720 (1), 19.3377 (4)	16.6174 (3), 4.8695 (1), 19.3375 (4)	16.6176 (4), 4.8671 (1), 19.3394 (5)	16.6155 (4), 4.8646 (1), 19.3384 (5)
β (°)	109.242 (2)	109.268 (2)	109.301 (3)	109.321 (3)
V (Å^3)	1478.23 (5)	1477.11 (5)	1476.25 (7)	1475.05 (7)
μ (mm^{-1})	0.82	0.82	0.82	0.82
Crystal size (mm)	0.47 × 0.41 × 0.12	0.45 × 0.4 × 0.14	0.45 × 0.4 × 0.14	0.45 × 0.41 × 0.14
Data collection				
T_{min}, T_{max}	0.764, 0.922	0.770, 0.923	0.769, 0.923	0.769, 0.923
No. of measured, independent and observed [$I > 2\sigma(I)$] reflections	4208, 1408, 1285	4202, 1407, 1279	4200, 1408, 1286	4196, 1406, 1302
R_{int}	0.023	0.023	0.023	0.023
$(\sin \theta/\lambda)_{max}$ (Å^{-1})	0.615	0.615	0.615	0.615
Refinement				
$R[F^2 > 2\sigma(F^2)]$, $wR(F^2)$, S	0.042, 0.119, 1.05	0.041, 0.118, 1.06	0.042, 0.118, 1.08	0.042, 0.117, 1.07
No. of reflections	1408	1407	1408	1406
$\Delta\rho_{max}$, $\Delta\rho_{min}$ (e Å^{-3})	0.20, −0.29	0.19, −0.27	0.21, −0.28	0.20, −0.26

Computer programs: *CrysAlis PRO* 1.171.42.49 (Rigaku OD, 2022), SHELXT 2014/4 (Sheldrick, 2014), *SHELXL* 2019/3 (Sheldrick, 2015), Olex2 1.5 (Dolomanov *et al.*, 2009).

Table S10 Experimental and crystallographic details for ETYSUC I sample crystal B (in 145-140 K temperature range). For all structures: $C_{12}H_{12}N_2{\cdot}C_4H_6O_4$, M_r = 302.32, monoclinic, *I*2/*a*, *Z* = 4. Experiments were carried out with Cu *K*α radiation using a New Xcalibur, EosS2. Gaussian, *CrysAlis PRO* 1.171.42.49 (Rigaku Oxford Diffraction, 2022) Absorption was corrected for by numerical methods absorption correction based on gaussian integration over a multifaceted crystal model Empirical absorption correction using spherical harmonics, implemented in SCALE3 ABSPACK scaling algorithm. Refinement was on 136 parameters. All H-atom parameters were refined.

	ETYSUC_145K_B	ETYSUC_140K_B
Crystal data		
Temperature (K)	145	140
a, *b*, *c* (Å)	16.6153 (5), 4.8619 (1), 19.3400 (5)	16.6128 (4), 4.8607 (1), 19.3367 (5)
β (°)	109.350 (3)	109.370 (3)
V (Å^3)	1474.07 (7)	1473.05 (7)
μ (mm^{-1})	0.82	0.82
Crystal size (mm)	0.45 × 0.41 × 0.14	0.45 × 0.41 × 0.14
Data collection		
T_{min}, T_{max}	0.769, 0.923	0.768, 0.923
No. of measured, independent and observed [$I > 2\sigma(I)$] reflections	4199, 1405, 1300	4200, 1405, 1293
R_{int}	0.023	0.022
$(\sin \theta/\lambda)_{max}$ (Å^{-1})	0.615	0.615
Refinement		
$R[F^2 > 2\sigma(F^2)]$, $wR(F^2)$, S	0.041, 0.114, 1.04	0.041, 0.113, 1.04
No. of reflections	1405	1405
$\Delta\rho_{max}$, $\Delta\rho_{min}$ (e Å^{-3})	0.20, −0.28	0.21, −0.28

Computer programs: *CrysAlis PRO* 1.171.42.49 (Rigaku OD, 2022), SHELXT 2014/4 (Sheldrick, 2014), *SHELXL* 2019/3 (Sheldrick, 2015), Olex2 1.5 (Dolomanov *et al.*, 2009).

Table S11 Experimental and crystallographic details for ETYFUM sample crystal A (in 300-150 K temperature range). For all structures: $C_{12}H_{12}N_2{\cdot}C_4H_4O_4$, M_r = 300.31, monoclinic, *I*2/*a*, *Z* = 4. Experiments were carried out with Cu *K*α radiation using a SuperNova, Single source at offset/far, Atlas. Gaussian, *CrysAlis PRO* 1.171.41.93a (Rigaku Oxford Diffraction, 2020) Absorption was corrected for by numerical methods absorption correction based on gaussian integration over a multifaceted crystal model Empirical absorption correction using spherical harmonics, implemented in SCALE3 ABSPACK scaling algorithm. All H-atom parameters were refined.

	etyfum_300k	ETYFUM_250K	etyfum_200k	etyfum_150k
Crystal data				
Temperature (K)	300	250	200	150
a, *b*, *c* (Å)	16.6911 (10), 4.8164 (3), 19.6669 (10)	16.6906 (9), 4.7903 (2), 19.6824 (9)	16.6840 (7), 4.7634 (2), 19.6972 (7)	16.6852 (7), 4.7457 (2), 19.6763 (7)
β (°)	109.032 (6)	109.367 (5)	109.674 (4)	109.949 (4)
V (Å^3)	1494.62 (16)	1484.62 (13)	1474.00 (11)	1464.54 (11)
μ (mm^{-1})	0.81	0.81	0.82	0.82
Crystal size (mm)	0.23 × 0.14 × 0.04	0.26 × 0.14 × 0.05	0.25 × 0.14 × 0.04	0.25 × 0.14 × 0.04
Data collection				
T_{min}, T_{max}	0.718, 1.000	0.599, 1.000	0.705, 1.000	0.697, 1.000
No. of measured, independent and observed [$I > 2\sigma(I)$] reflections	2839, 1440, 1119	2821, 1432, 1131	2815, 1426, 1187	2787, 1418, 1195
R_{int}	0.024	0.025	0.024	0.022
$(\sin \theta/\lambda)_{max}$ (Å^{-1})	0.619	0.617	0.618	0.618
Refinement				
$R[F^2 > 2\sigma(F^2)]$, $wR(F^2)$, S	0.037, 0.108, 1.02	0.035, 0.099, 1.06	0.034, 0.093, 1.05	0.035, 0.100, 1.05
No. of reflections	1440	1432	1426	1418
No. of parameters	133	133	133	132
No. of restraints	0	0	0	0
$\Delta\rho_{max}$, $\Delta\rho_{min}$ (e Å^{-3})	0.12, −0.15	0.15, −0.13	0.19, −0.15	0.14, −0.25

Computer programs: *CrysAlis PRO* 1.171.41.93a (Rigaku OD, 2020), SHELXT 2014/4 (Sheldrick, 2014), SHELXT (Sheldrick, 2015), *SHELXL* 2014/7 (Sheldrick, 2015), Olex2 1.5 (Dolomanov *et al.*, 2009).

Table S12 Experimental and crystallographic details for ETYFUM sample crystal A (in 130-100 K temperature range). For all structures: $C_{12}H_{12}N_2{\cdot}C_4H_4O_4$, M_r = 300.31, monoclinic, *I*2/*a*, *Z* = 4. Experiments were carried out with Cu *K*α radiation using a SuperNova, Single source at offset/far, Atlas. Gaussian, *CrysAlis PRO* 1.171.41.93a (Rigaku Oxford Diffraction, 2020) Absorption was corrected for by numerical methods absorption correction based on gaussian integration over a multifaceted crystal model Empirical absorption correction using spherical harmonics, implemented in SCALE3 ABSPACK scaling algorithm. All H-atom parameters were refined.

	etyfum_130k	etyfum_100k
Crystal data		
Temperature (K)	130	100
a, *b*, *c* (Å)	16.6728 (5), 4.7388 (1), 19.6680 (6)	16.6633 (6), 4.7294 (2), 19.6488 (7)
β (°)	110.024 (3)	110.091 (4)
V (Å^3)	1460.01 (7)	1454.24 (10)
μ (mm^{-1})	0.83	0.83
Crystal size (mm)	0.25 × 0.15 × 0.04	0.25 × 0.15 × 0.04
Data collection		
T_{min}, T_{max}	0.699, 1.000	0.699, 1.000
No. of measured, independent and observed [$I > 2\sigma(I)$] reflections	2783, 1415, 1211	2766, 1405, 1210
R_{int}	0.022	0.021
$(\sin \theta/\lambda)_{max}$ (Å^{-1})	0.618	0.618
Refinement		
$R[F^2 > 2\sigma(F^2)]$, $wR(F^2)$, S	0.035, 0.097, 1.04	0.032, 0.087, 1.04
No. of reflections	1415	1405
No. of parameters	132	132
No. of restraints	0	1
$\Delta\rho_{max}$, $\Delta\rho_{min}$ (e Å^{-3})	0.17, −0.24	0.19, −0.20

Computer programs: *CrysAlis PRO* 1.171.41.93a (Rigaku OD, 2020), SHELXT 2014/4 (Sheldrick, 2014), SHELXT (Sheldrick, 2015), *SHELXL* 2014/7 (Sheldrick, 2015), Olex2 1.5 (Dolomanov *et al.*, 2009).

Table S13 Experimental and crystallographic details for ETYFUM I sample crystal B (in 300-185 K temperature range). For all structures: $C_{12}H_{12}N_2{\cdot}C_4H_4O_4$, M_r = 300.31, monoclinic, *I*2/*a*, *Z* = 4. Experiments were carried out with Cu *K*α radiation using a New Xcalibur, EosS2. Gaussian, *CrysAlis PRO* 1.171.41.93a (Rigaku Oxford Diffraction, 2020) Absorption was corrected for by numerical methods absorption correction based on gaussian integration over a multifaceted crystal model Empirical absorption correction using spherical harmonics, implemented in SCALE3 ABSPACK scaling algorithm. Refinement was on 132 parameters. All H-atom parameters were refined.

	etyfum_300k_b	etyfum_200k_b	etyfum_190k_b	etyfum_185k_b
Crystal data				
Temperature (K)	300	200	190	185
a, *b*, *c* (Å)	16.6920 (4), 4.8145 (1), 19.6756 (5)	16.6798 (5), 4.7618 (1), 19.6808 (7)	16.6841 (7), 4.7564 (2), 19.6818 (8)	16.6800 (7), 4.7547 (2), 19.6846 (8)
β (°)	109.059 (3)	109.657 (4)	109.740 (5)	109.773 (5)
V (Å^3)	1494.53 (7)	1472.07 (8)	1470.09 (11)	1469.11 (11)
μ (mm^{-1})	0.81	0.82	0.82	0.82
Crystal size (mm)	0.41 × 0.29 × 0.16	0.41 × 0.29 × 0.16	0.41 × 0.29 × 0.16	0.41 × 0.29 × 0.16
Data collection				
T_{min}, T_{max}	0.791, 0.901	0.788, 0.900	0.787, 0.900	0.787, 0.900
No. of measured, independent and observed [$I > 2\sigma(I)$] reflections	4197, 1428, 1219	4148, 1412, 1264	4152, 1407, 1255	4157, 1408, 1260
R_{int}	0.023	0.027	0.026	0.026
$(\sin \theta/\lambda)_{max}$ (Å^{-1})	0.615	0.615	0.615	0.615
Refinement				
$R[F^2 > 2\sigma(F^2)]$, $wR(F^2)$, S	0.043, 0.128, 1.06	0.041, 0.117, 1.05	0.041, 0.118, 1.03	0.042, 0.121, 1.05
No. of reflections	1428	1412	1407	1408
No. of restraints	0	0	0	0
$\Delta\rho_{max}$, $\Delta\rho_{min}$ (e Å^{-3})	0.17, −0.19	0.17, −0.24	0.18, −0.21	0.18, −0.24

Computer programs: *CrysAlis PRO* 1.171.41.93a (Rigaku OD, 2020), SHELXT 2018/2 (Sheldrick, 2018), *SHELXL* 2019/3 (Sheldrick, 2015), Olex2 1.5 (Dolomanov *et al.*, 2009).

Table S14 Experimental and crystallographic details for ETYFUM I sample crystal B (in 180-170 K temperature range). For all structures: $C_{12}H_{12}N_2{\cdot}C_4H_4O_4$, M_r = 300.31, monoclinic, $I2/a$, Z = 4. Experiments were carried out with Cu $K\alpha$ radiation using a New Xcalibur, EosS2. Gaussian, *CrysAlis PRO* 1.171.41.93a (Rigaku Oxford Diffraction, 2020) Absorption was corrected for by numerical methods absorption correction based on gaussian integration over a multifaceted crystal model Empirical absorption correction using spherical harmonics, implemented in SCALE3 ABSPACK scaling algorithm. Refinement was on 132 parameters. All H-atom parameters were refined.

	etyfum_180k_b	etyfum_175k_b	etyfum_170k_b
Crystal data			
Temperature (K)	180	175	170
a, b, c (Å)	16.6837 (6), 4.7513 (2), 19.6812 (8)	16.6837 (7), 4.7500 (2), 19.6845 (8)	16.6847 (7), 4.7475 (2), 19.6847 (9)
β (°)	109.784 (4)	109.814 (5)	109.844 (5)
V (Å^3)	1468.03 (11)	1467.60 (11)	1466.65 (12)
μ (mm^{-1})	0.82	0.82	0.82
Crystal size (mm)	0.41 × 0.29 × 0.16	0.41 × 0.29 × 0.16	0.41 × 0.29 × 0.16
Data collection			
T_{min}, T_{max}	0.786, 0.900	0.787, 0.900	0.787, 0.900
No. of measured, independent and observed [$I > 2\sigma(I)$] reflections	4151, 1408, 1279	4152, 1406, 1267	4135, 1403, 1260
R_{int}	0.023	0.025	0.024
(sin θ/λ)$_{max}$ (Å^{-1})	0.615	0.615	0.615
Refinement			
$R[F^2 > 2\sigma(F^2)]$, $wR(F^2)$, S	0.041, 0.118, 1.05	0.041, 0.119, 1.05	0.041, 0.120, 1.07
No. of reflections	1408	1406	1403
No. of restraints	0	0	1
$\Delta\rho_{max}$, $\Delta\rho_{min}$ (e Å^{-3})	0.20, −0.23	0.19, −0.23	0.18, −0.22

Computer programs: *CrysAlis PRO* 1.171.41.93a (Rigaku OD, 2020), SHELXT 2018/2 (Sheldrick, 2018), *SHELXL* 2019/3 (Sheldrick, 2015), Olex2 1.5 (Dolomanov *et al.*, 2009).

Table S15 Experimental and crystallographic details for ETYFUM I sample crystal B (in 165-150 K temperature range). For all structures: $C_{12}H_{12}N_2{\cdot}C_4H_4O_4$, M_r = 300.31, monoclinic, *I*2/*a*, *Z* = 4. Experiments were carried out with Cu *K*α radiation using a New Xcalibur, EosS2. Gaussian, *CrysAlis PRO* 1.171.41.93a (Rigaku Oxford Diffraction, 2020) Absorption was corrected for by numerical methods absorption correction based on gaussian integration over a multifaceted crystal model Empirical absorption correction using spherical harmonics, implemented in SCALE3 ABSPACK scaling algorithm. Refinement was on 132 parameters. All H-atom parameters were refined.

	etyfum_165k_b	etyfum_160k_b	etyfum_155k_b	etyfum_150k_b
Crystal data				
Temperature (K)	165	160	155	150
a, *b*, *c* (Å)	16.6819 (6), 4.7422 (2), 19.6945 (8)	16.6868 (6), 4.7394 (1), 19.6945 (7)	16.6821 (8), 4.7392 (2), 19.6849 (9)	16.6834 (8), 4.7362 (2), 19.6927 (10)
β (°)	109.915 (4)	109.936 (4)	109.948 (5)	109.981 (6)
V (Å^3)	1464.84 (11)	1464.21 (9)	1462.91 (12)	1462.37 (13)
μ (mm^{-1})	0.82	0.82	0.82	0.82
Crystal size (mm)	0.41 × 0.29 × 0.16	0.41 × 0.29 × 0.16	0.41 × 0.29 × 0.16	0.41 × 0.29 × 0.16
Data collection				
T_{min}, T_{max}	0.787, 0.900	0.787, 0.900	0.787, 0.899	0.787, 0.899
No. of measured, independent and observed [$I > 2\sigma(I)$] reflections	4144, 1399, 1263	4147, 1397, 1271	4149, 1397, 1272	4133, 1396, 1277
R_{int}	0.026	0.025	0.026	0.023
$(\sin \theta/\lambda)_{max}$ (Å^{-1})	0.615	0.615	0.615	0.615
Refinement				
$R[F^2 > 2\sigma(F^2)]$, $wR(F^2)$, S	0.040, 0.116, 1.05	0.041, 0.117, 1.05	0.042, 0.122, 1.06	0.041, 0.118, 1.04
No. of reflections	1399	1397	1397	1396
No. of restraints	0	1	0	1
$\Delta\rho_{max}$, $\Delta\rho_{min}$ (e Å^{-3})	0.18, −0.24	0.20, −0.22	0.18, −0.24	0.20, −0.23

Computer programs: *CrysAlis PRO* 1.171.41.93a (Rigaku OD, 2020), SHELXT 2018/2 (Sheldrick, 2018), *SHELXL* 2019/3 (Sheldrick, 2015), Olex2 1.5 (Dolomanov *et al.*, 2009).

Table S16 Experimental and crystallographic details for ETYFUM I sample crystal B (in 145-140 K temperature range). For all structures: $C_{12}H_{12}N_2{\cdot}C_4H_4O_4$, M_r = 300.31, monoclinic, *I*2/*a*, *Z* = 4. Experiments were carried out with Cu *K*α radiation using a New Xcalibur, EosS2. Gaussian, *CrysAlis PRO* 1.171.41.93a (Rigaku Oxford Diffraction, 2020) Absorption was corrected for by numerical methods absorption correction based on gaussian integration over a multifaceted crystal model Empirical absorption correction using spherical harmonics, implemented in SCALE3 ABSPACK scaling algorithm. Refinement was on 132 parameters. All H-atom parameters were refined.

	etyfum_145k_b	etyfum_140k_b
Crystal data		
Temperature (K)	145	140
a, *b*, *c* (Å)	16.6876 (7), 4.7329 (2), 19.6859 (9)	16.6842 (6), 4.7293 (1), 19.6920 (7)
β (°)	110.002 (5)	110.036 (4)
V ($Å^3$)	1461.02 (12)	1459.75 (9)
μ (mm^{-1})	0.82	0.83
Crystal size (mm)	0.41 × 0.29 × 0.16	0.41 × 0.29 × 0.16
Data collection		
T_{min}, T_{max}	0.849, 0.930	0.788, 0.899
No. of measured, independent and observed [$I > 2\sigma(I)$] reflections	4130, 1394, 1262	4110, 1385, 1257
R_{int}	0.024	0.025
$(\sin \theta/\lambda)_{max}$ ($Å^{-1}$)	0.615	0.615
Refinement		
$R[F^2 > 2\sigma(F^2)]$, $wR(F^2)$, S	0.041, 0.118, 1.05	0.040, 0.115, 1.03
No. of reflections	1394	1385
No. of restraints	1	1
$\Delta\rho_{max}$, $\Delta\rho_{min}$ (e $Å^{-3}$)	0.19, −0.22	0.20, −0.23

Computer programs: *CrysAlis PRO* 1.171.41.93a (Rigaku OD, 2020), SHELXT 2018/2 (Sheldrick, 2018), *SHELXL* 2019/3 (Sheldrick, 2015), Olex2 1.5 (Dolomanov *et al.*, 2009).

S2.2. Principal axis strain

S2.2.1. Compressibility

Table S17 Principal axis strain calculation details for ETYSUC I.

Input data							
p **(GPa)**	**σ*p*** **(GPa)**	***a*** **(Å)**	***b*** **(Å)**	***c*** **(Å)**	***α*** **(º)**	***β*** **(º)**	***γ*** **(º)**
0.0001	1.0	16.6711	4.9272	19.3744	90.0	108.512	90.0
0.64	2.0	16.6	4.7781	19.218	90.0	110.32	90.0
0.94	2.0	16.56	4.7226	19.187	90.0	110.99	90.0
1.41	2.0	16.55	4.6266	19.089	90.0	111.96	90.0
1.95	2.0	16.53	4.5612	19.065	90.0	112.63	90.0
2.46	2.0	16.48	4.5205	19.083	90.0	113.24	90.0
2.76	2.0	16.469	4.4857	19.042	90.0	113.63	90.0
2.87	2.0	16.462	4.4828	19.038	90.0	113.66	90.0
2.9	2.0	16.446	4.46879	19.038	90.0	113.81	90.0

Principal axis strain calculation output									
			**Direction**			**Empirical parameters**			
**Axes**	***K*** **(TPa^{-1})**	**σ*K*(TPa^{-1})**	***a***	***B***	***c***	ε_0	λ	p_c	***N***
X_1	25.6159	1.1113	-0.0	1.0	-0.0	82.4118	-82.3848	-1.3713	0.001
X_2	19.7591	0.6398	0.733	0.0	0.6802	41.4967	-41.4979	-0.9784	0.0014
X_3	-5.3924	0.1802	0.8024	0.0	-0.5968	-16.503	16.4846	-2.2521	0.0014
V	49.9663	3.8144							

Birch-Murnaghan coefficients							
	B_0 (GPa)	σB_0 (GPa)	V_0 (Å^3)	σV_0 (Å^3)	B'	$\sigma B'$	p_c (GPa)
2nd	12.961	0.6008	1502.3701	6.6472	4.0	n/a	0.0
3rd	8.3106	1.244	1510.0306	6.1941	10.3143	2.2785	0.0

Table S18 Principal axis strain calculation details for ETYSUC I.- *continuation*

**Compressibility**						
p **(GPa)**	K_1 **(TPa⁻¹)**	K_2 **(TPa⁻¹)**	K_3 **(TPa⁻¹)**	σK_1 **(TPa⁻¹)**	σK_2 **(TPa⁻¹)**	σK_3 **(TPa⁻¹)**
0.0001	61.981	59.0442	-10.0525	10.9663	8.7952	0.879
0.64	42.2782	35.7235	-7.831	2.0259	1.6761	0.3184
0.94	36.7959	30.1441	-7.0959	2.1976	1.6229	0.3144
1.41	30.5838	24.2196	-6.1864	1.4638	0.9289	0.2184
1.95	25.6159	19.7591	-5.3924	1.1113	0.6398	0.1802
2.46	22.2094	16.832	-4.8095	2.0303	1.1859	0.3371
2.76	20.5982	15.4831	-4.522	2.6393	1.517	0.4481
2.87	20.0645	15.0411	-4.4251	2.8529	1.6292	0.4885
2.9	19.9237	14.925	-4.3993	2.91	1.6589	0.4994
**Change in length**						
p **(GPa)**	X_1 **(%)**	X_2 **(%)**	X_3 **(%)**	$X_{1,calc}$ **(%)**	$X_{2,calc}$ **(%)**	$X_{3,calc}$ **(%)**
0.0001	-0.0	0.0	0.0	0.0172	0.004	-0.0036
0.64	-3.0718	-2.8327	0.538	-3.2385	-2.9041	0.5626
0.94	-4.2385	-3.8496	0.7577	-4.4208	-3.8873	0.7862
1.41	-6.2861	-5.3452	1.1213	-5.9953	-5.1547	1.0973
1.95	-7.7023	-6.2932	1.4307	-7.5047	-6.334	1.409
2.46	-8.5921	-7.1438	1.7027	-8.7201	-7.2631	1.6686
2.76	-9.358	-7.7711	1.8252	-9.3616	-7.7472	1.8085
2.87	-9.4221	-7.8397	1.8123	-9.5853	-7.9151	1.8577
2.9	-9.7319	-8.0743	1.8584	-9.6452	-7.9601	1.8709
**Comment**						
Pressure ESD on SCXRD measurement at ambient conditions was set to 1, while data for high-pressure experiments were set with ESD 2 (due to less precise determination of lattice constants for crystal inside DAC).						

Table S19 Principal axis strain calculation details for ETYSUC I'.

Input data

p (GPa)	σp (GPa)	a (Å)	b (Å)	c (Å)	α (º)	β (º)	γ (º)
2.97	2.0	16.437	4.472	19.05	90.0	113.74	90.0
3.17	3.0	16.44	4.472	19.05	90.0	114.0	90.0
3.29	2.0	16.429	4.4214	19.02	90.0	114.32	90.0
3.59	3.0	16.45	4.398	18.94	90.0	114.5	90.0
3.73	4.0	16.46	4.3839	18.94	90.0	114.3	90.0

Principal axis strain calculation output

			Direction			Empirical parameters			
Axes	K (TPa^{-1})	σK(TPa^{-1})	a	B	c	ε_0	λ	p_c	N
X_1	17.242	32.2219	0.7339	-0.0	0.6793	13.715	-13.7285	2.7221	0.0007
X_2	28.1852	36.2845	0.0	1.0	0.0	5.8727	-5.8314	1.0553	0.0107
X_3	-2.0193	3.175	0.8071	0.0	-0.5904	-2.0996	2.1025	2.9563	0.0003
V	41.8634	3.5358							

Birch-Murnaghan coefficients

	B_0 (GPa)	σB_0 (GPa)	V_0 (Å^3)	σV_0 (Å^3)	B'	$\sigma B'$	p_c (GPa)
2nd	8.9022	3.6865	1593.5691	104.0552	4.0	n/a	0.0
3rd	0.0876	5355.2529	2065.8565	1262030.4864	98.2663	5584750.039	0.0
3rd with Pc	0.0552	5155.7042	1305.3427	11139.3335	10878.1703	1012616565.8631	2.9

Compressibility

p (GPa)	K_1 (TPa^{-1})	K_2 (TPa^{-1})	K_3 (TPa^{-1})	σK_1 (TPa^{-1})	σK_2 (TPa^{-1})	σK_3 (TPa^{-1})
2.97	39.4766	32.8414	-49.1791	201.1809	104.9338	817.6605
3.17	21.8579	29.767	-3.1528	47.355	24.9134	8.6564
3.29	17.242	28.1852	-2.0193	32.2219	36.2845	3.175
3.59	11.2854	24.8828	-1.0635	21.9243	36.5771	1.3067
3.73	9.7188	23.594	-0.8712	37.2457	88.7431	1.6564

Change in length

p (GPa)	X_1 (%)	X_2 (%)	X_3 (%)	$X_{1,calc}$ (%)	$X_{2,calc}$ (%)	$X_{3,calc}$ (%)
2.97	-0.0	0.0	0.0	0.0154	0.059	-0.0002
3.17	-0.3391	0.0	0.1576	-0.5636	-0.5661	0.1849
3.29	-0.8834	-1.1379	0.2314	-0.7961	-0.9136	0.2149
3.59	-1.3516	-1.6684	0.2709	-1.2114	-1.7076	0.2582
3.73	-1.0974	-1.9894	0.231	-1.3579	-2.0467	0.2716

Comment

Due to the narrow range of pressure in which Form I' was obtained, limited also by hydrostatic limit of used PTM (3.8-4 GPa) not enough experimental points were collected to enable reliable calculation of compressibility. Additionally, measurement at 3.47 GPa had very large ESDs of unit-cell parameters length and was removed from the analysis. Moreover, poorer quality of data (large ESDs and short measurement used just to measure lattice constants) were assigned higher ESD on pressure to lower their weight in calculation of principle axes strain. The ESD values of compressibility (higher than the values itself) deems analysis of NLC in ETYSUC I' unreliable. Pressure ESDs were assigned in following manner: 2 for high-pressure measurements of higher quality; 3 for measurements where only lattice constants were determined; 4 for data collected under highest pressure where crystal strain affected quality of collected data.

Table S20 Principal axis strain calculation details for combined ETYSUC I and ETYSUC I' data.

Input data							
***p* (GPa)**	**σ*p* (GPa)**	***a* (Å)**	***b* (Å)**	***c* (Å)**	***α* (º)**	***β* (º)**	***γ* (º)**
0.0001	1.0	16.6711	4.9272	19.3744	90.0	108.512	90.0
0.64	2.0	16.6	4.7781	19.218	90.0	110.32	90.0
0.94	2.0	16.56	4.7226	19.187	90.0	110.99	90.0
1.41	2.0	16.55	4.6266	19.089	90.0	111.96	90.0
1.95	2.0	16.53	4.5612	19.065	90.0	112.63	90.0
2.46	2.0	16.48	4.5205	19.083	90.0	113.24	90.0
2.76	2.0	16.469	4.4857	19.042	90.0	113.63	90.0
2.87	2.0	16.462	4.4828	19.038	90.0	113.66	90.0
2.9	2.0	16.446	4.46879	19.038	90.0	113.81	90.0
2.97	2.0	16.437	4.472	19.05	90.0	113.74	90.0
3.17	3.0	16.44	4.472	19.05	90.0	114.0	90.0
3.29	2.0	16.429	4.4214	19.02	90.0	114.32	90.0
3.47	5.0	16.44	4.4104	18.97	90.0	114.0	90.0
3.59	3.0	16.45	4.398	18.94	90.0	114.5	90.0
3.73	4.0	16.46	4.3839	18.94	90.0	114.3	90.0

Principal axis strain calculation output									
			**Direction**			**Empirical parameters**			
**Axes**	***K* (TPa⁻¹)**	**σ*K*(TPa⁻¹)**	***a***	***B***	***c***	***ε₀***	***λ***	***p$_c$***	***N***
X_1	21.2018	1.7965	0.0	1.0	0.0	0.2728	-0.2743	-0.9751	0.2208
X_2	15.6503	1.0355	0.7457	-0.0	0.6663	0.2449	-0.2606	-0.6963	0.1721
X_3	-4.2055	0.4365	0.7806	0.0	-0.625	-19.1577	19.1446	-1.9105	0.001
V	45.8877	3.6073							

Birch-Murnaghan coefficients							
	**B_0 (GPa)**	**σB_0 (GPa)**	**V_0 (Å³)**	**σV_0 (Å³)**	***B′***	**σ*B′***	**p_c (GPa)**
2nd	13.2868	0.461	1501.1236	5.5617	4.0	n/a	0.0
3rd	9.8121	1.2384	1507.9092	6.523	7.578	1.5102	0.0

Comment

Pressure ESDs were assigned as described in Comment sections of Tables S18 and S19, and additionally data point at 3.47 GPa was included (with ESD of 5 due to very low quality of the data measurement).

Table S21 Principal axis strain calculation details for combined ETYSUC I and ETYSUC I' data.- *continuation*

**Compressibility**						
p **(GPa)**	K_1 **(TPa^{-1})**	K_2 **(TPa^{-1})**	K_3 **(TPa^{-1})**	σK_1 **(TPa^{-1})**	σK_2 **(TPa^{-1})**	σK_3 **(TPa^{-1})**
0.0001	61.7506	60.5068	-10.5123	12.6694	10.1323	2.3098
0.64	41.6783	35.2751	-7.8773	2.105	1.5901	0.3122
0.94	36.4967	29.8296	-7.0491	2.3486	1.5538	0.3797
1.41	30.7597	24.2025	-6.0523	1.7089	0.975	0.3592
1.95	26.2372	20.0355	-5.2065	0.8991	0.4986	0.2371
2.46	23.1489	17.3154	-4.5996	1.1914	0.7242	0.2899
2.76	21.6868	16.0615	-4.3044	1.631	0.9536	0.3942
2.87	21.2018	15.6503	-4.2055	1.7965	1.0355	0.4365
2.9	21.0738	15.5421	-4.1793	1.8413	1.0574	0.4482
2.97	20.7819	15.296	-4.1194	1.945	1.1076	0.4756
3.17	19.9963	14.638	-3.9574	2.2331	1.2442	0.5537
3.29	19.5565	14.2722	-3.8662	2.3988	1.3209	0.6
3.47	18.9367	13.7598	-3.737	2.6361	1.4288	0.6678
3.59	18.5476	13.4401	-3.6556	2.7867	1.496	0.7116
3.73	18.1162	13.0872	-3.5649	2.9547	1.5698	0.7613
**Change in length**						
p **(GPa)**	X_1 **(%)**	X_2 **(%)**	X_3 **(%)**	$X_{1,calc}$ **(%)**	$X_{2,calc}$ **(%)**	$X_{3,calc}$ **(%)**
0.0001	-0.0	0.0	0.0	0.0085	0.0026	-0.0059
0.64	-3.0718	-2.8327	0.538	-3.2053	-2.9035	0.5743
0.94	-4.2385	-3.8496	0.7577	-4.374	-3.8751	0.7978
1.41	-6.2861	-5.3452	1.1213	-5.9457	-5.1347	1.1045
1.95	-7.7023	-6.2932	1.4307	-7.4773	-6.3213	1.4073
2.46	-8.5921	-7.1438	1.7027	-8.7329	-7.27	1.6567
2.76	-9.358	-7.7711	1.8252	-9.4048	-7.7701	1.7902
2.87	-9.4221	-7.8397	1.8123	-9.6407	-7.9445	1.837
2.9	-9.7319	-8.0743	1.8584	-9.7041	-7.9913	1.8496
2.97	-9.6608	-7.9825	1.8117	-9.8506	-8.0993	1.8786
3.17	-9.6608	-8.2985	1.9934	-10.2583	-8.3985	1.9594
3.29	-10.7855	-8.8102	2.0976	-10.4956	-8.5719	2.0063
3.47	-11.0312	-8.5107	1.8042	-10.8419	-8.8242	2.0747
3.59	-11.3088	-9.1881	2.1026	-11.0668	-8.9873	2.1191
3.73	-11.6251	-8.9103	2.0042	-11.3234	-9.173	2.1696

Table S22 Principal axis strain calculation details for ETYFUM cited after Patyk-Kaźmierczak and Kaźmierczak (Patyk-Kaźmierczak & Kaźmierczak, 2024).

**Input data**							
p **(GPa)**	**σ*p* (GPa)**	***a*** **(Å)**	***b*** **(Å)**	***c*** **(Å)**	***α*** **(º)**	***β*** **(º)**	***γ*** **(º)**
0.0001	1.0	16.6937	4.8165	19.6871	90.0	109.083	90.0
0.08	3.0	16.68	4.7884	19.65	90.0	109.1	90.0
0.15	3.0	16.72	4.7728	19.7	90.0	110.1	90.0
0.29	3.0	16.7	4.702	19.65	90.0	110.1	90.0
0.35	2.0	16.686	4.6593	19.75	90.0	110.45	90.0
0.53	3.0	16.73	4.5924	19.74	90.0	110.9	90.0
0.79	2.0	16.758	4.5205	19.83	90.0	111.8	90.0
0.88	3.0	16.72	4.4815	19.84	90.0	111.79	90.0
1.25	2.0	16.758	4.3755	19.955	90.0	113.05	90.0
1.33	3.0	16.79	4.3174	19.94	90.0	113.2	90.0
1.85	2.0	16.808	4.246	20.027	90.0	114.38	90.0
2.44	2.0	16.836	4.1496	20.12	90.0	115.18	90.0
3.0	2.0	16.874	4.056	20.17	90.0	116.14	90.0
3.58	3.0	16.89	3.993	20.19	90.0	115.9	90.0

**Principal axis strain calculation output**									
			**Direction**			**Empirical parameters**			
**Axes**	***K*** **(TPa^{-1})**	**σ*K*(TPa^{-1})**	***a***	***B***	***c***	***ε₀***	***λ***	***p*$_c$**	***N***
X_1	68.013	2.2443	-0.0	1.0	-0.0	152.8631	-152.8235	-1.3111	0.0009
X_2	27.8274	1.8883	0.7788	0.0	0.6273	73.4964	-73.5008	-0.9098	0.0006
X_3	-24.3616	0.9754	0.7292	0.0	-0.6843	-75.3736	75.3449	-1.6144	0.0008
V	54.0133	4.1208							

**Birch-Murnaghan coefficients**							
	***B*$_0$ (GPa)**	**σ*B*$_0$ (GPa)**	***V*$_0$ (Å^3)**	**σ*V*$_0$ (Å^3)**	***B′***	**σ*B′***	***p*$_c$ (GPa)**
2nd	11.2529	0.4323	1487.528	5.3724	4.0	n/a	0.0
3rd	7.3076	0.8266	1498.1606	5.3212	9.1543	1.4172	0.0

Table S23 Principal axis strain calculation details for ETYFUM cited after Patyk-Kaźmierczak and Kaźmierczak (Patyk-Kaźmierczak & Kaźmierczak, 2024)-*continuation*

**Compressibility**						
p **(GPa)**	K_1 (TPa^{-1})	K_2 (TPa^{-1})	K_3 (TPa^{-1})	σK_1 (TPa^{-1})	σK_2 (TPa^{-1})	σK_3 (TPa^{-1})
0.0001	108.9387	51.9632	-36.2692	9.7938	11.1588	5.9181
0.08	102.6872	47.7713	-34.5602	6.8996	7.3013	4.5406
0.15	97.7719	44.618	-33.1902	5.0508	4.987	3.5899
0.29	89.2302	39.415	-30.7521	2.8531	2.4037	2.2456
0.35	86.01	37.539	-29.8136	2.4141	1.9347	1.8529
0.53	77.6084	32.8489	-27.3129	2.1512	1.7655	1.1769
0.79	68.013	27.8274	-24.3616	2.2443	1.8883	0.9754
0.88	65.2219	26.429	-23.4833	2.1957	1.8422	0.9686
1.25	55.8073	21.9041	-20.4522	1.6967	1.4246	0.9118
1.33	54.1184	21.1222	-19.8969	1.57	1.3254	0.8936
1.85	45.2235	17.1447	-16.9126	1.2383	0.9978	0.8976
2.44	38.1164	14.1268	-14.4533	2.0496	1.3592	1.2517
3.0	33.1695	12.1047	-12.7005	2.9582	1.83	1.6958
3.58	29.2396	10.5419	-11.2834	3.7424	2.2297	2.1213
**Change in length**						
p **(GPa)**	X_1 (%)	X_2 (%)	X_3 (%)	$X_{1,calc}$ (%)	$X_{2,calc}$ (%)	$X_{3,calc}$ (%)
0.0001	-0.0	0.0	0.0	0.0921	0.0008	-0.0591
0.08	-0.5851	-0.199	-0.082	-0.7528	-0.3972	0.2237
0.15	-0.9114	-1.1396	0.7471	-1.4542	-0.7203	0.4608
0.29	-2.4055	-1.3324	0.5671	-2.7614	-1.3071	0.9079
0.35	-3.317	-1.562	1.003	-3.287	-1.5378	1.0896
0.53	-4.7607	-1.997	1.3788	-4.7569	-2.1694	1.6031
0.79	-6.3336	-2.8143	2.2615	-6.6445	-2.9546	2.2734
0.88	-7.1958	-2.9072	2.1795	-7.2439	-3.1987	2.4886
1.25	-9.5709	-4.1065	3.3915	-9.4739	-4.0876	3.2989
1.33	-10.892	-4.2173	3.5308	-9.9135	-4.2597	3.4602
1.85	-12.5335	-5.4633	4.5663	-12.4826	-5.2474	4.4131
2.44	-14.78	-6.1948	5.4055	-14.9292	-6.1642	5.3346
3.0	-16.9922	-7.1966	6.2631	-16.9188	-6.8958	6.0928
3.58	-18.4969	-6.7909	6.2034	-18.7238	-7.5504	6.7867

S2.2.2. Thermal expansion

Table S24 Details on calculation of linear coefficient of thermal expansion for ETYSUC.

Input data							
T **(K)**	**σ*T*** **(K)**	***a*** **(Å)**	***b*** **(Å)**	***c*** **(Å)**	***α*** **(°)**	***β*** **(°)**	***γ*** **(°)**
99.9	0.3	16.6142	4.8463	19.3219	90.0	109.542	90.0
140.05	0.1	16.6128	4.8607	19.3367	90.0	109.37	90.0
145.05	0.1	16.6153	4.8619	19.34	90.0	109.35	90.0
150.05	0.1	16.6155	4.8646	19.3384	90.0	109.321	90.0
150.1	0.14	16.6244	4.8694	19.3334	90.0	109.283	90.0
155.05	0.1	16.6176	4.8671	19.3394	90.0	109.301	90.0
160.05	0.1	16.6174	4.8695	19.3375	90.0	109.268	90.0
165.05	0.1	16.6187	4.872	19.3377	90.0	109.242	90.0
170.0	0.14	16.6197	4.8747	19.3362	90.0	109.217	90.0
175.0	0.14	16.6216	4.877	19.3388	90.0	109.193	90.0
180.0	0.14	16.6256	4.8792	19.3393	90.0	109.164	90.0
185.05	0.1	16.6268	4.881	19.3397	90.0	109.141	90.0
190.0	0.1	16.6279	4.8825	19.3411	90.0	109.105	90.0
199.95	0.1	16.6313	4.8871	19.3441	90.0	109.05	90.0
200.1	0.3	16.6436	4.8875	19.3528	90.0	109.051	90.0
250.0	0.14	16.65	4.9071	19.3547	90.0	108.799	90.0
300.0	0.14	16.6688	4.9269	19.3733	90.0	108.522	90.0
300.1	0.8	16.6655	4.9261	19.3776	90.0	108.507	90.0

Linear coefficient of thermal expansion					
			**Direction**		
**Axes**	***α*** **(MK^{-1})**	**σ*α*(MK^{-1})**	***a***	***b***	***c***
X_1	-16.4719	0.6737	0.7898	0.0	-0.6133
X_2	79.8202	1.6417	0.7416	0.0	0.6709
X_3	84.3082	1.2081	-0.0	1.0	-0.0
V	149.0838	2.2056			

Table S25 Details on calculation of linear coefficient of thermal expansion for ETYSUC.-*continuation*

Change in length						
T **(K)**	X_1 (%)	X_2 (%)	X_3 (%)	$X_{1,calc}$ (%)	$X_{2,calc}$ (%)	$X_{3,calc}$ (%)
99.9	-0.0	-0.0	0.0	-0.0162	-0.0771	-0.0194
140.05	-0.0781	0.2528	0.2967	-0.0823	0.2434	0.3191
145.05	-0.074	0.2932	0.3214	-0.0905	0.2833	0.3613
150.05	-0.0935	0.3237	0.3769	-0.0988	0.3232	0.4034
150.1	-0.0991	0.3802	0.4755	-0.0989	0.3236	0.4038
155.05	-0.0959	0.3562	0.4283	-0.107	0.3631	0.4456
160.05	-0.1208	0.3904	0.4776	-0.1153	0.403	0.4877
165.05	-0.1318	0.4264	0.5289	-0.1235	0.4429	0.5299
170.0	-0.1473	0.4556	0.5843	-0.1316	0.4824	0.5716
175.0	-0.1496	0.4976	0.6315	-0.1399	0.5223	0.6138
180.0	-0.1535	0.5461	0.6766	-0.1481	0.5622	0.6559
185.05	-0.163	0.579	0.7134	-0.1564	0.6025	0.6985
190.0	-0.1782	0.6303	0.7442	-0.1646	0.6421	0.7402
199.95	-0.1939	0.716	0.8383	-0.181	0.7215	0.8241
200.1	-0.134	0.7742	0.8465	-0.1812	0.7227	0.8254
250.0	-0.265	1.1088	1.2467	-0.2634	1.121	1.2461
300.0	-0.3306	1.5538	1.6493	-0.3458	1.5201	1.6676
300.1	-0.338	1.573	1.6331	-0.3459	1.5209	1.6685

Table S26 Details on calculation of linear coefficient of thermal expansion for ETYFUM (140-300 K).

Input data							
T **(K)**	**σ*T*** **(K)**	***a*** **(Å)**	***b*** **(Å)**	***c*** **(Å)**	***α*** **(°)**	***β*** **(°)**	***γ*** **(°)**
140.0	0.14	16.6842	4.72926	19.692	90.0	110.036	90.0
145.05	0.1	16.6876	4.73285	19.6859	90.0	110.002	90.0
150.05	0.1	16.6834	4.7362	19.6927	90.0	109.948	90.0
155.05	0.1	16.6821	4.7392	19.6849	90.0	109.958	90.0
160.05	0.1	16.6868	4.73945	19.6945	90.0	109.935	90.0
165.05	0.1	16.6819	4.74216	19.6945	90.0	109.915	90.0
170.0	0.14	16.6847	4.74752	19.6847	90.0	109.845	90.0
175.1	0.2	16.6837	4.75	19.6845	90.0	109.814	90.0
180.1	0.2	16.6837	4.75134	19.6812	90.0	109.784	90.0
185.1	0.14	16.68	4.75471	19.6846	90.0	109.774	90.0
190.0	0.14	16.6841	4.7564	19.6818	90.0	109.74	90.0
200.05	0.1	16.6798	4.76176	19.6808	90.0	109.657	90.0
200.1	0.3	16.684	4.7634	19.6972	90.0	109.674	90.0
249.9	0.2	16.6906	4.7903	19.6824	90.0	109.367	90.0
299.6	0.8	16.6911	4.8164	19.6669	90.0	109.032	90.0
299.95	0.1	16.692	4.81447	19.6756	90.0	109.059	90.0

Linear coefficient of thermal expansion					
			**Direction**		
**Axes**	***α*** **(MK^{-1})**	**σ*α*(MK^{-1})**	***a***	***b***	***c***
X_1	-38.5846	0.7756	0.7199	0.0	-0.6941
X_2	75.1745	0.9384	0.7826	0.0	0.6225
X_3	111.4434	1.2062	-0.0	1.0	-0.0
V	148.9456	1.2946			

Table S27 Details on calculation of linear coefficient of thermal expansion for ETYFUM (140-300 K).-*continuation*

Change in length						
T (K)	X_1 (%)	X_2 (%)	X_3 (%)	$X_{1,calc}$ (%)	$X_{2,calc}$ (%)	$X_{3,calc}$ (%)
140.0	-0.0	0.0	0.0	-0.0078	-0.0109	0.0216
145.05	-0.0363	0.0473	0.0759	-0.0273	0.027	0.0779
150.05	-0.0545	0.1092	0.1466	-0.0466	0.0646	0.1336
155.05	-0.073	0.074	0.21	-0.0659	0.1022	0.1893
160.05	-0.0475	0.1401	0.2152	-0.0852	0.1398	0.2451
165.05	-0.0753	0.1512	0.2724	-0.1045	0.1774	0.3008
170.0	-0.1349	0.2223	0.3854	-0.1236	0.2146	0.356
175.1	-0.1569	0.2569	0.4376	-0.1432	0.2529	0.4128
180.1	-0.1843	0.2867	0.4658	-0.1625	0.2905	0.4685
185.1	-0.1913	0.2952	0.5367	-0.1818	0.3281	0.5242
190.0	-0.2082	0.3439	0.5722	-0.2007	0.3649	0.5788
200.05	-0.2732	0.4308	0.6848	-0.2395	0.4405	0.6908
200.1	-0.2085	0.4639	0.7193	-0.2397	0.4409	0.6914
249.9	-0.4148	0.8289	1.2824	-0.4318	0.8152	1.2464
299.6	-0.6591	1.2089	1.8256	-0.6236	1.1889	1.8003
299.95	-0.6161	1.1987	1.7855	-0.625	1.1915	1.8042

Table S28 Details on calculation of linear coefficient of thermal expansion for ETYFUM (100-150 K).

Input data							
T **(K)**	**σ*T*** **(K)**	***a*** **(Å)**	***b*** **(Å)**	***c*** **(Å)**	***α*** **(°)**	***β*** **(°)**	***γ*** **(°)**
99.9	0.3	16.6633	4.7294	19.6488	90.0	110.091	90.0
130.0	0.3	16.6728	4.7388	19.668	90.0	110.024	90.0
150.1	0.2	16.6852	4.7457	19.6763	90.0	109.949	90.0

Linear coefficient of thermal expansion					
			**Direction**		
**Axes**	***α*** **(MK^{-1})**	**σ*α*(MK^{-1})**	***a***	***b***	***c***
X_1	9.7492	0.2798	0.888	0.0	-0.4599
X_2	62.3276	1.8497	0.6811	0.0	0.7322
X_3	68.5383	0.623	-0.0	1.0	-0.0
V	141.0501	2.2422			

Change in length						
T **(K)**	X_1 **(%)**	X_2 **(%)**	X_3 **(%)**	$X_{1,calc}$ **(%)**	$X_{2,calc}$ **(%)**	$X_{3,calc}$ **(%)**
99.9	-0.0	-0.0	0.0	0.001	-0.0066	-0.0022
130.0	0.0328	0.1646	0.1986	0.0303	0.181	0.2041
150.1	0.0489	0.3129	0.3441	0.0499	0.3063	0.3418

Table S29 Details on calculation of linear coefficient of thermal expansion for ETYFUM (all data).

Input data							
T **(K)**	**σ*T*** **(K)**	***a*** **(Å)**	***b*** **(Å)**	***c*** **(Å)**	***α*** **(°)**	***β*** **(°)**	***γ*** **(°)**
99.9	0.3	16.6633	4.7294	19.6488	90.0	110.091	90.0
130.0	0.3	16.6728	4.7388	19.668	90.0	110.024	90.0
140.0	0.14	16.6842	4.72926	19.692	90.0	110.036	90.0
145.05	0.1	16.6876	4.73285	19.6859	90.0	110.002	90.0
150.05	0.1	16.6834	4.7362	19.6927	90.0	109.948	90.0
150.1	0.2	16.6852	4.7457	19.6763	90.0	109.949	90.0
155.05	0.1	16.6821	4.7392	19.6849	90.0	109.958	90.0
160.05	0.1	16.6868	4.73945	19.6945	90.0	109.935	90.0
165.05	0.1	16.6819	4.74216	19.6945	90.0	109.915	90.0
170.0	0.14	16.6847	4.74752	19.6847	90.0	109.845	90.0
175.1	0.2	16.6837	4.75	19.6845	90.0	109.814	90.0
180.1	0.2	16.6837	4.75134	19.6812	90.0	109.784	90.0
185.1	0.14	16.68	4.75471	19.6846	90.0	109.774	90.0
190.0	0.14	16.6841	4.7564	19.6818	90.0	109.74	90.0
200.05	0.1	16.6798	4.76176	19.6808	90.0	109.657	90.0
200.1	0.3	16.684	4.7634	19.6972	90.0	109.674	90.0
249.9	0.2	16.6906	4.7903	19.6824	90.0	109.367	90.0
299.6	0.8	16.6911	4.8164	19.6669	90.0	109.032	90.0
299.95	0.1	16.692	4.81447	19.6756	90.0	109.059	90.0

Linear coefficient of thermal expansion					
			**Direction**		
**Axes**	***α*** **(MK^{-1})**	**σ*α*(MK^{-1})**	***a***	***b***	***c***
X_1	-34.1933	2.7182	0.8161	0.0	-0.578
X_2	73.9405	1.0864	0.7338	0.0	0.6794
X_3	104.4302	4.3975	-0.0	1.0	-0.0
V	145.4296	2.79			

Table S30 Details on calculation of linear coefficient of thermal expansion for ETYFUM (all data).- *continuation*

Change in length						
T (K)	X_1 (%)	X_2 (%)	X_3 (%)	$X_{1,calc}$ (%)	$X_{2,calc}$ (%)	$X_{3,calc}$ (%)
99.9	-0.0	-0.0	0.0	0.2356	-0.0555	-0.3461
130.0	0.0328	0.1646	0.1986	0.1327	0.167	-0.0318
140.0	0.1184	0.2617	-0.003	0.0985	0.241	0.0726
145.05	0.1098	0.2814	0.0729	0.0812	0.2783	0.1254
150.05	0.0739	0.3611	0.1437	0.0641	0.3153	0.1776
150.1	0.0489	0.3129	0.3441	0.0639	0.3156	0.1781
155.05	0.0615	0.3198	0.207	0.047	0.3522	0.2298
160.05	0.0836	0.3893	0.2123	0.0299	0.3892	0.282
165.05	0.0525	0.4038	0.2694	0.0128	0.4262	0.3342
170.0	0.0037	0.4642	0.3824	-0.0041	0.4628	0.3859
175.1	-0.0188	0.4994	0.4346	-0.0216	0.5005	0.4392
180.1	-0.0444	0.5274	0.4628	-0.0387	0.5375	0.4914
185.1	-0.0552	0.5397	0.5337	-0.0558	0.5744	0.5436
190.0	-0.0684	0.5848	0.5693	-0.0725	0.6107	0.5948
200.05	-0.1349	0.6734	0.6819	-0.1069	0.685	0.6997
200.1	-0.0742	0.7105	0.7163	-0.107	0.6854	0.7003
249.9	-0.2734	1.0691	1.2794	-0.2773	1.0536	1.2203
299.6	-0.5156	1.4476	1.8226	-0.4473	1.4211	1.7393
299.95	-0.4737	1.4384	1.7826	-0.4485	1.4236	1.743

S2.2.3. Compressibility capacity

Table S31 Compressibility capacity for ETYSUC I and ETYSUC all data, as well as for the selected NLC materials.

NLC material	χ_K (%)	Investigated pressure range	Ref. (original data report)	Ref. (χ_K calculation)
ETYFUM	6.846	0.1 MPa-3.58 GPa	(Patyk-Kaźmierczak & Kaźmierczak, 2024)	(Patyk-Kaźmierczak & Kaźmierczak, 2024)
CPOS-1	5.401	1.02-2.32 GPa	(Zhao *et al.*, 2020)	(Patyk-Kaźmierczak & Kaźmierczak, 2024)
$Zn[Au(CN)_2]_2$ I	5.374	0.35-1.75 GPa	(Cairns *et al.*, 2013)	(Patyk-Kaźmierczak & Kaźmierczak, 2024)
$Ag_3[Co(CN)_6]$ II	5.251	0.19-7.65 GPa	(Goodwin *et al.*, 2008)	(Patyk-Kaźmierczak & Kaźmierczak, 2024)
UNIRUF II	5.056	2.01-5.51 GPa	(Yeung *et al.*, 2017)	(Patyk-Kaźmierczak & Kaźmierczak, 2024)
JOHGIX	4.389	0.1MPa-2.42 GPa	(Woodall *et al.*, 2013)	(Patyk-Kaźmierczak & Kaźmierczak, 2024)
$InH(DBC)_2$	3.72	0.1 MPa-0.53 GPa	(Zeng *et al.*, 2017)	(Patyk-Kaźmierczak & Kaźmierczak, 2024)
$Zn[Au(CN)_2]_2$ II	3.602	2.04-14.44 GPa	(Cairns *et al.*, 2013)	(Patyk-Kaźmierczak & Kaźmierczak, 2024)
MIL-53(Al)	2.74	0.1 MPa-1.8 GPa	(Jiang *et al.*, 2022)	(Patyk-Kaźmierczak & Kaźmierczak, 2024)
MAGVOG I	2.544	0.1MPa-0.9 GPa	(Cai & Katrusiak, 2014)	(Patyk-Kaźmierczak & Kaźmierczak, 2024)
BIUREA II	2.285	0.62-2.81 GPa	(Bull *et al.*, 2019)	(Patyk-Kaźmierczak & Kaźmierczak, 2024)
**ETYSUC (all data)**	**2.188**	**0.1 MPa-3.73 GPa**	**This work**	**This work**
water@CPOS-1	2.113	0.58-2.53 GPa	(Zhao *et al.*, 2020)	(Patyk-Kaźmierczak & Kaźmierczak, 2024)
POWSID	2.039	0.1MPa-2.28 GPa	(Shepherd *et al.*, 2012)	(Patyk-Kaźmierczak & Kaźmierczak, 2024)
**ETYSUC I**	**1.875**	**0.1 MPa-2.90 GPa**	**This work**	**This work**
QAXMEH yellow	1.225	0.54-2.84 GPa	(Harty *et al.*, 2015)	(Patyk-Kaźmierczak & Kaźmierczak, 2024)
$Ag_3[Co(CN)_6]$ I	1.164	0.1 MPa-0.157 GPa	(Goodwin *et al.*, 2008)	(Patyk-Kaźmierczak & Kaźmierczak, 2024)
IZIYOI hp	1.059	1.36-2.39 GPa	(Szafrański, 2020)	(Patyk-Kaźmierczak & Kaźmierczak, 2024)
SULBAC	0.984	0.91-2.83 GPa	(Shephard *et al.*, 2022)	(Patyk-Kaźmierczak & Kaźmierczak, 2024)

S2.3. Wine rack motif deformation

Table S32 Parameters of a triangle formed by centroids acting as hinge points in structure of ETYSUC I and ETYSUC I' (compressed sample).

	p **(GPa)**	$\boldsymbol{d_1}$ **(Å)**	$\boldsymbol{d_2}$ **(Å)**	***h*** **(Å)**	***φ*** **(°)**
ETYSUC I	0.0001	3.902	4.927	3.0262	78.29
	0.64	3.875	4.778	3.05104	76.12
	0.94	3.877	4.723	3.0748	75.05
	1.41	3.867	4.627	3.09885	73.48
	1.95	3.849	4.561	3.10063	72.67
	2.46	3.84	4.521	3.10406	72.13
	2.76	3.837	4.486	3.11303	71.55
	2.87	3.857	4.483	3.13907	71.05
	2.9	3.829	4.469	3.10966	71.39
ETYSUC I'	2.97	3.816	4.482	3.08877	71.92
	3.29	3.837	4.421	3.13616	70.36
	3.47	3.819	4.41	3.11779	70.55
	3.73	3.81	4.384	3.11639	70.24

Table S33 Functions fitted to parameters of a the triangle formed by centroids acting as hinge points in ETYSUC I and ETYSUC I' (compressed sample).

	**Parameter (y)**	**Fitted function ($y = a - bc^x$)**	$\boldsymbol{R^2}$
ETYSUC I	d_1	$d_1 = 3.81134 + 0.08986 * 0.66742^p$	0.87807
	d_2	$d_2 = 4.30351 + 0.62602 * 0.64044^p$	0.99825
	h	$h = 3.1444 - 0.12014 * 0.57997^p$	0.91758
	φ	$\varphi = 68.74507 + 9.61032 * 0.63332^p$	0.99370
ETYSUC I'		**Fitted function ($y = a + bx$)**	
	d_1	$d_1 = 3.85845 - 0.01128 * p$	0.09607
	d_2	$d_2 = 4.85057 - 0.12669 * p$	0.94993
	h	$h = 3.00949 + 0.03129 * p$	0.26157
	φ	$\varphi = 77.80826 - 2.09235 * p$	0.73588

Table S34 Parameters of a triangle formed by centroids acting as hinge points in structure of ETYSUC I (cooled sample).

T **(K)**	$\boldsymbol{d_1}$ **(Å)**	$\boldsymbol{d_2}$ **(Å)**	***h*** **(Å)**	***φ*** **(°)**
300.1	3.904	4.928	3.02818	78.27
250	3.901	4.907	3.03315	77.93
200.1	3.904	4.888	3.04446	77.51
150.1	3.897	4.865	3.04474	77.24
99.9	3.898	4.846	3.05357	76.86

Table S35 Functions fitted to parameters of a the triangle formed by centroids acting as hinge points in ETYSUC I (cooled sample).

Parameter (y)	Fitted function ($y = a + bx$)	R^2
d_1	$d_1 = 3.8944 + 0.0000319832 * T$	0.59822
d_2	$d_2 = 4.80443 + 0.000411751 * T$	0.99924
h	$h = 3.06575 - 0.000124657 * T$	0.94925
φ	$\varphi = 76.15858 + 0.00702 * T$	0.99685

Table S36 Parameters of a triangle formed by centroids acting as hinge points in structure of ETYFUM (cooled sample).

T (K)	d_1 (Å)	d_2 (Å)	h (Å)	φ (°)
299.6	3.892	4.816	3.05729	76.46
249.9	3.891	4.79	3.06657	75.98
200.1	3.89	4.763	3.07537	75.52
150.1	3.886	4.746	3.07739	75.27
130	3.884	4.739	3.07767	75.18
99.9	3.881	4.729	3.07756	75.07

Table S37 Functions fitted to parameters of a the triangle formed by centroids acting as hinge points in ETYFUM (cooled sample).

	Parameter (y)	Fitted function ($y = a + bx$)	R^2
ETYFUM (150-300 K)	d_1	$d_1 = 3.88117 + 0.0000381489 * T$	0.87076
	d_2	$d_2 = 4.67179 + 0.000475557 * T$	0.99048
	h	$h = 3.10033 - 0.000138588 * T$	0.93973
	φ	$\varphi = 73.98875 + 0.00809 * T$	0.98147
ETYFUM-lt (100-150 K)	d_1	$d_1 = 3.87105 + 0.0000996068 * T$	1
	d_2	$d_2 = 4.69517 + 0.000338141 * T$	0.99983
	h	$h = 3.0779 - 0.00000687842 * T$	0.26883
	φ	$\varphi = 74.67197 + 0.00396 * T$	0.99677

S3. Figures

S3.1. Sample crystals

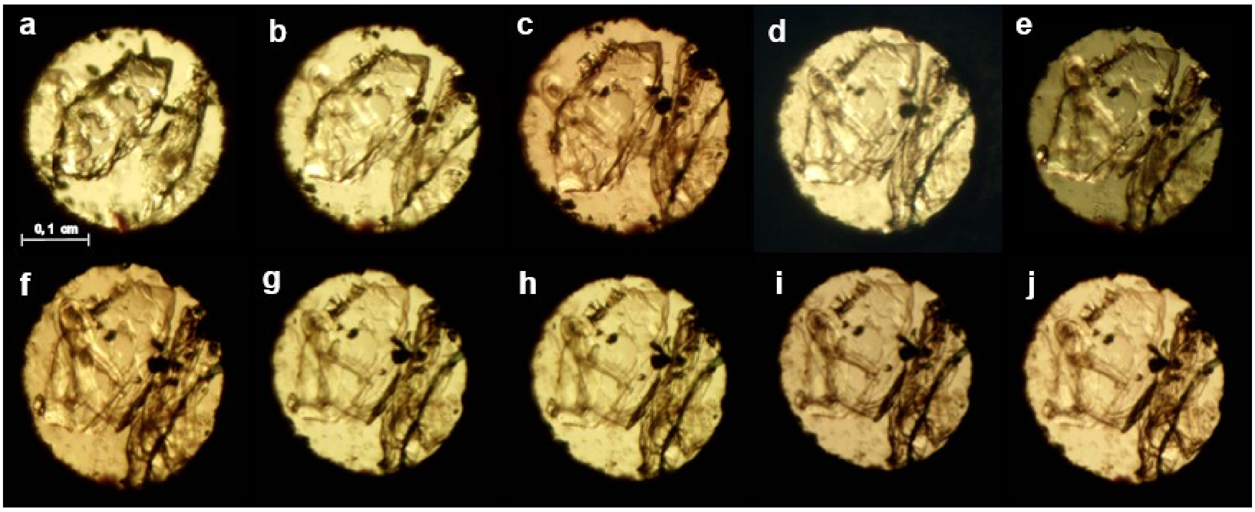

Figure S1 Single crystal of ETYSUC mounted in diamond anvil cell at 295 K and (a) 0.1 MPa, (b) 0.40(2), (c) 0.64(2), (d) 0.94(2), (e) 1.41(2), (f) 1.95(2), (g) 2.46(2), (h) 2.92(2), (i) 3.47(2), (j) 3.73 (j) GPa. A cellulose fibre used to fix the position of the sample crystal during the measurement is visible in the bottom, right part of the chamber, next to the gasket edge.

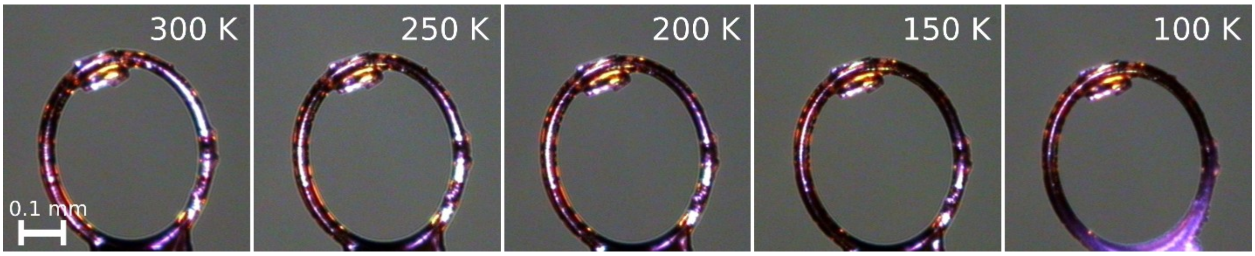

Figure S2 ETYSUC sample crystal A measured in 100-300 K temperature range. Scale is included in the bottom left corner of the first segment, while temperature is listed in the top right corner of each picture.

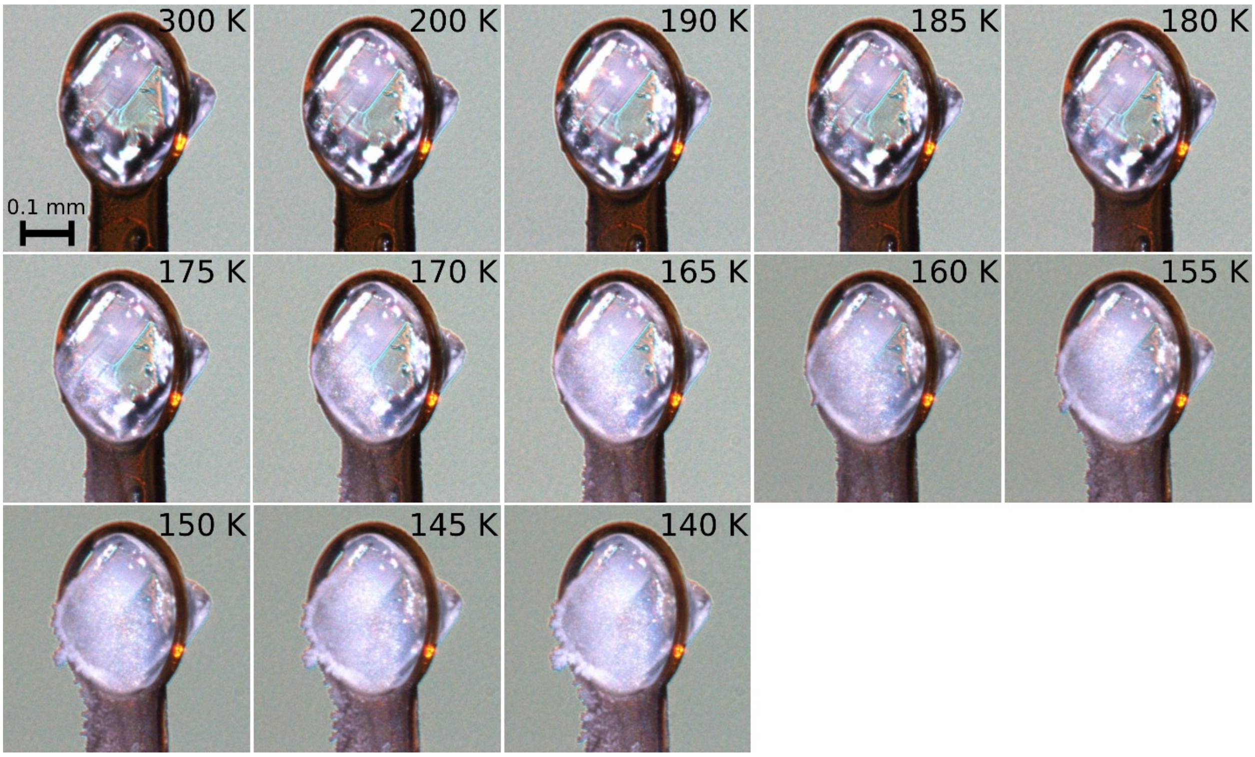

Figure S3 ETYSUC sample crystal B measured in 140-300 K temperature range. Scale is included in the bottom left corner of the first segment, while temperature is listed in the top right corner of each picture.

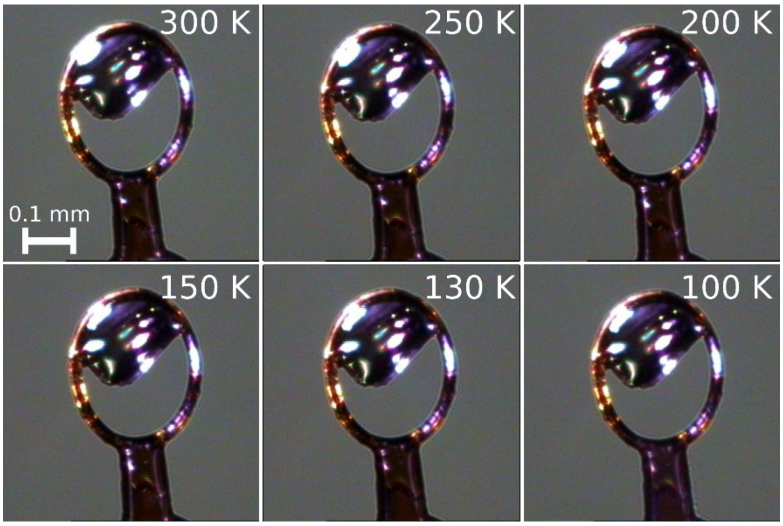

Figure S4 ETYFUM sample crystal A measured in 100-300 K temperature range. Scale is included in the bottom left corner of the first segment, while temperature is listed in the top right corner of each picture.

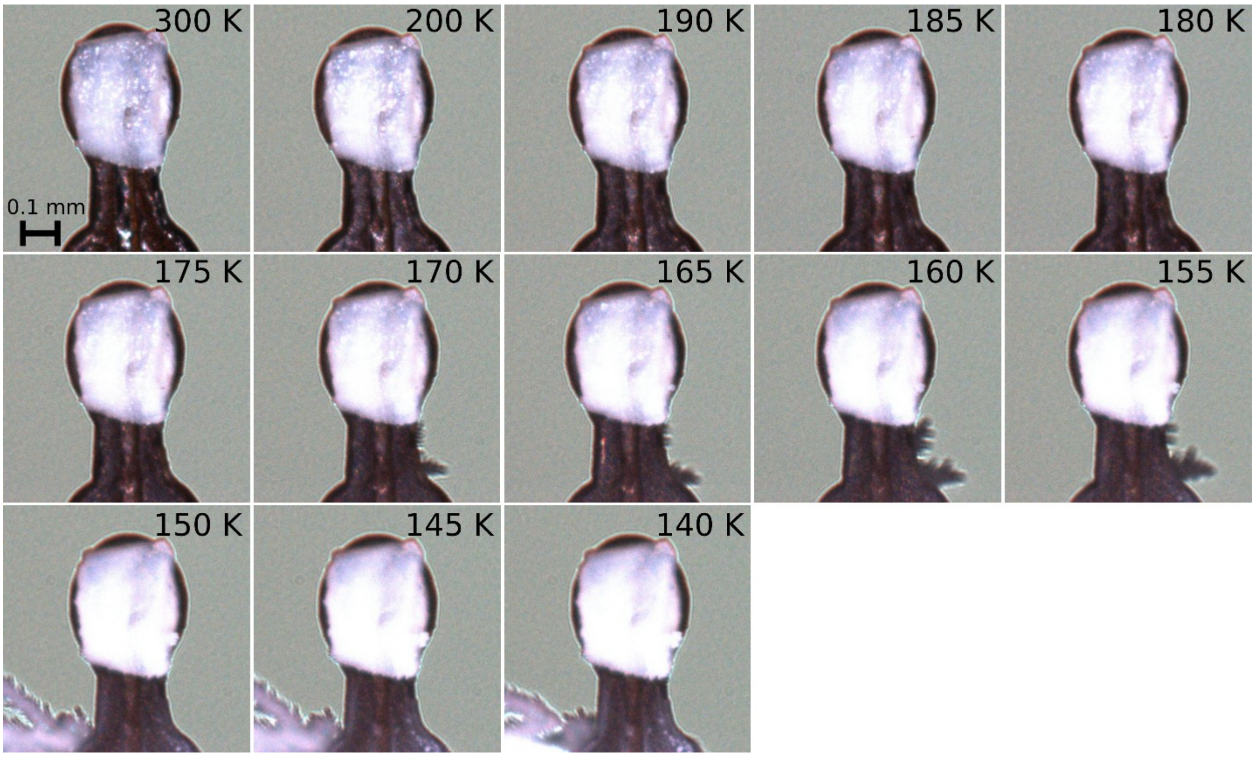

Figure S5 ETYFUM sample crystal B measured in 140-300 K temperature range. Scale is included in the bottom left corner of the first segment, while temperature is listed in the top right corner of each picture.

S3.2. PXRD patterns

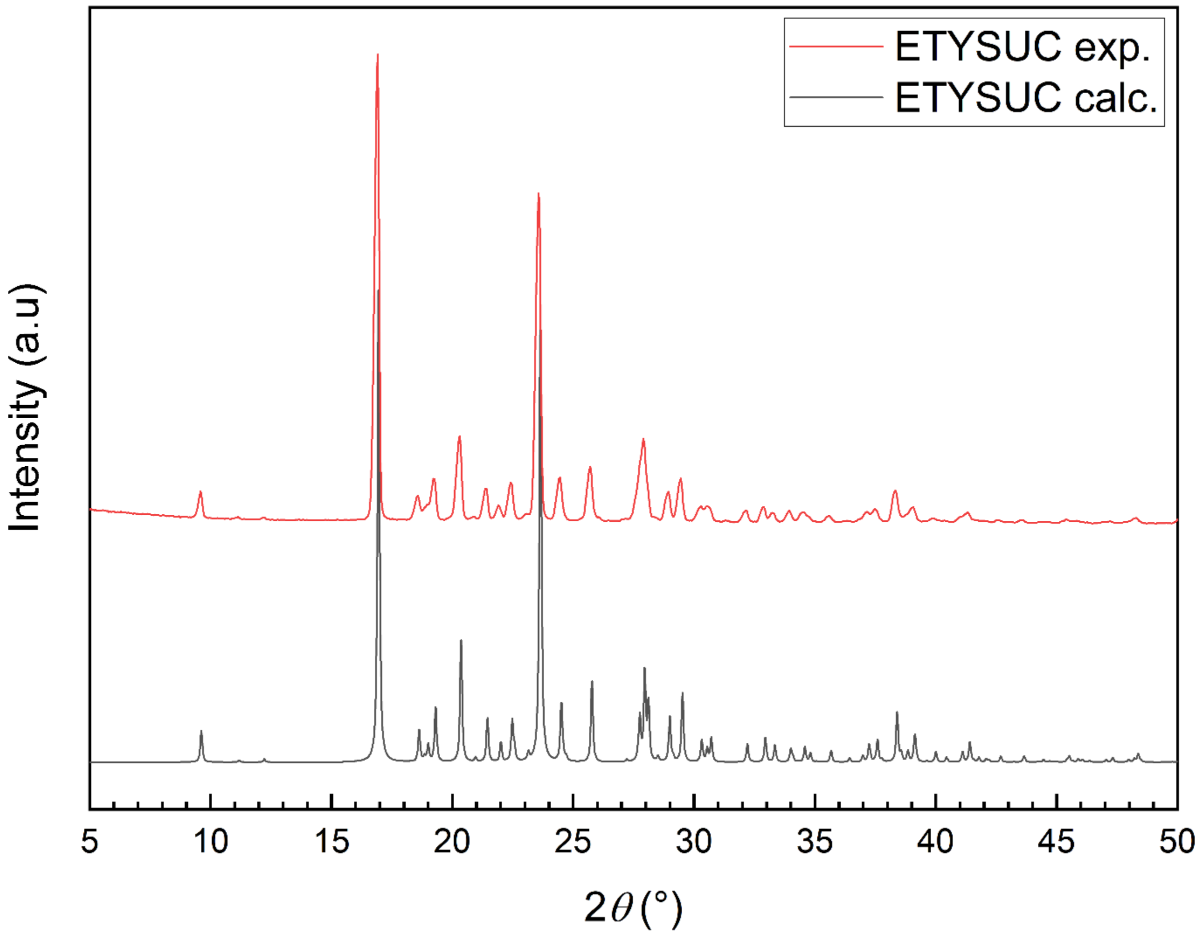

Figure S6 Experimental PXRD pattern for ETYSUC sample obtained after solvent-assisted ball milling (red), alongside PXRD pattern calculated with Mercury, based on crystal structure at 295 K/0.1 MPa (black).

S3.3. Principal axis strain

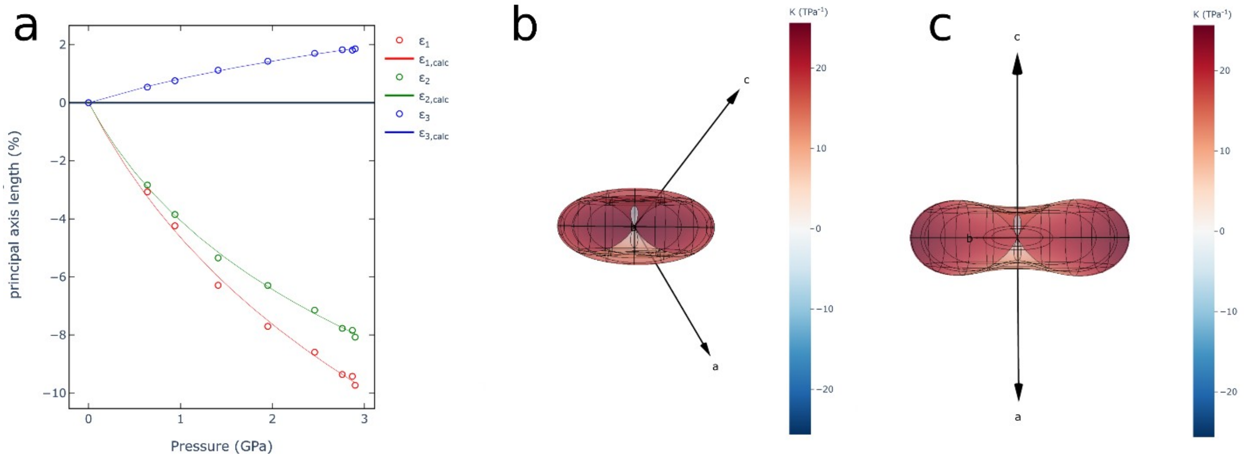

Figure S7 Relative change in length of the principal axes in function of pressure (a), and compressibility indicatrix plots (b, c) representing compressibility tensors for ETYSUC I (0.1 MPa-2.90 GPa pressure range). The indicatrix plots are shown along crystallographic axis *b* (b), and along direction [101] (c). Positive and negative linear compressibility is marked in red and blue, respectively, in the indicatrix plots.

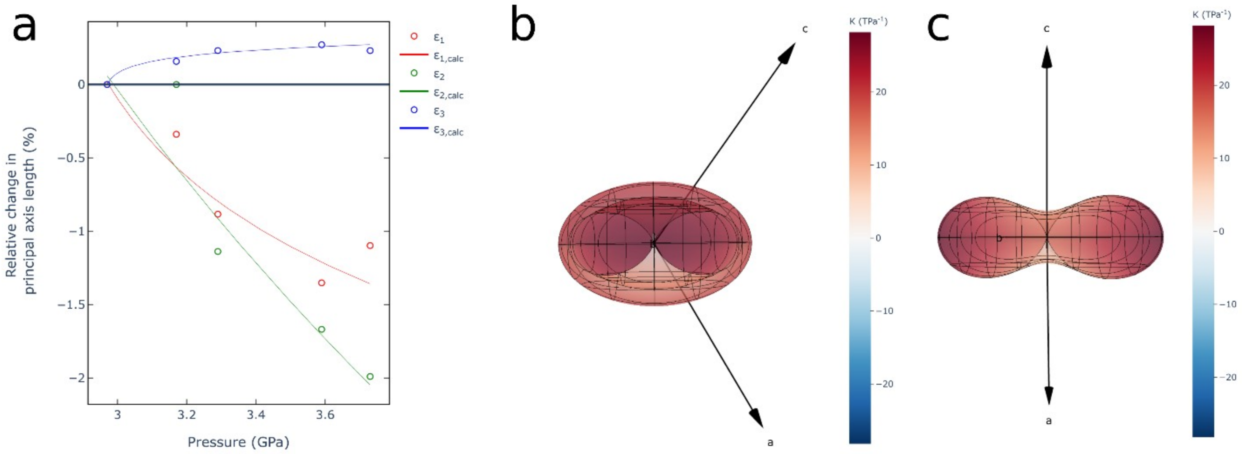

Figure S8 Relative change in length of the principal axes in function of pressure (a), and compressibility indicatrix plots (b, c) representing compressibility tensors for ETYSUC I' (2.97 MPa-3.73 GPa pressure range). The indicatrix plots are shown along crystallographic axis *b* (b), and along direction [101] (c). Positive and negative linear compressibility is marked in red and blue, respectively, in the indicatrix plots.

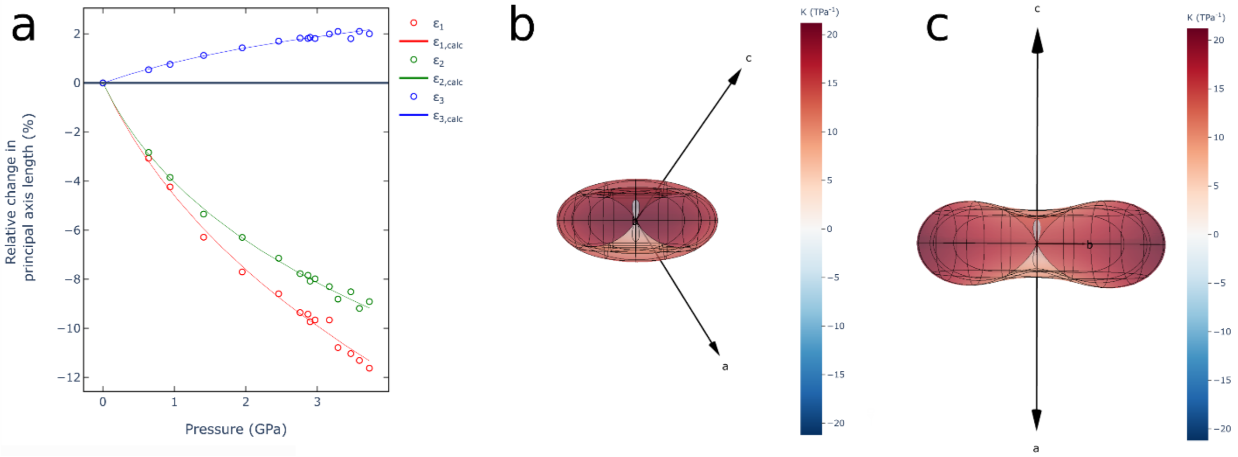

Figure S9 Relative change in length of the principal axes in function of pressure (a), and compressibility indicatrix plots (b,c) representing compressibility tensors for ETYSUC I and I' (0.1 MPa-3.73 GPa pressure range). The indicatrix plots are shown along crystallographic axis *b* (b), and along direction [101] (c). Positive and negative linear compressibility is marked in red and blue, respectively, in the indicatrix plots.

S3.4. Molecular aggregation

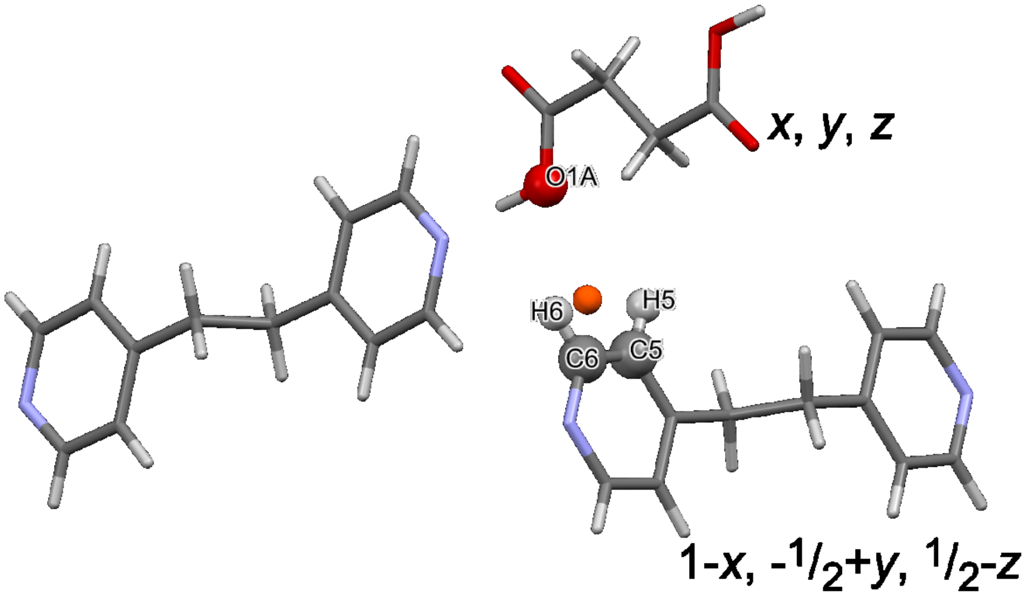

Figure S10 Positioning of SUC and ETY molecules in ETYSUC structure (at 0.1 MPa/300 K) showing oxygen atom O1A of SUC interlocked between carbon atoms C5 and C6 of ETY (all atoms involved in the 'interlock' are shown in 'ball and stick' mode). A centroid calculated for atoms O1A, C5, C6 is marked in orange. Symmetry codes are listed next to molecules of interest.

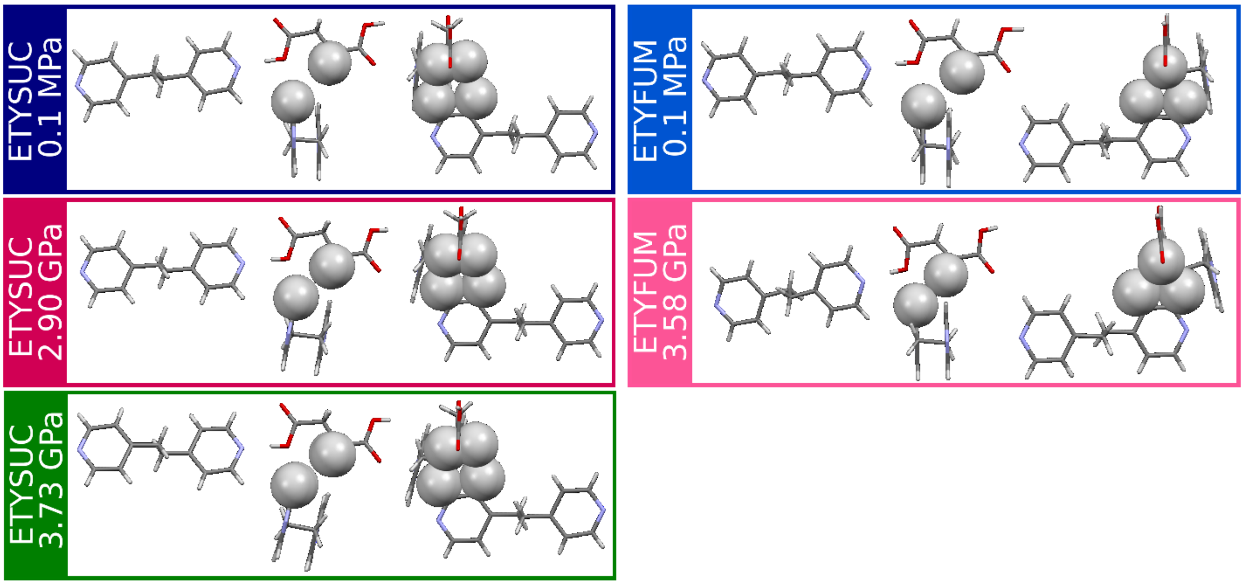

Figure S11 The arrangement of ETY and SUC molecules (left), and ETY and FUM molecules (right) at the *hinge point,* shown at different pressure (all at room temperature). The hydrogen atoms of ETY, SUC and FUM molecules that are involved are shown as full spheres to show steric hindrance between the molecules.

S3.5. Negative Linear Compressibility

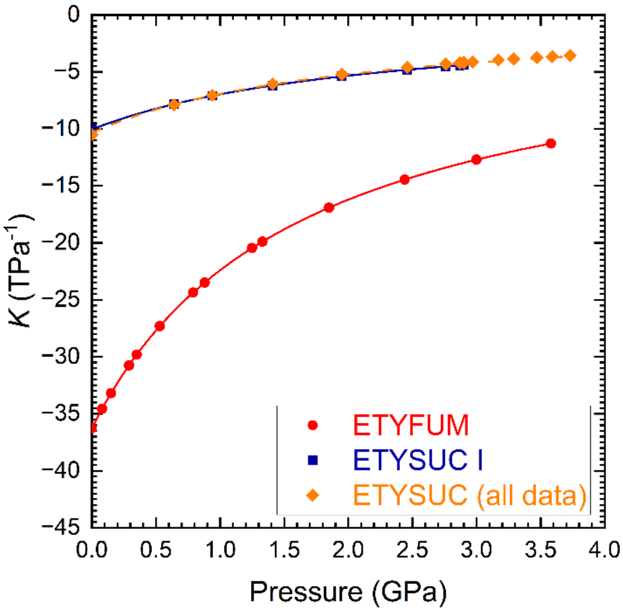

Figure S12 Pressure-dependence of compressibility *K* calculated using PASCal (Cliffe & Goodwin, 2012; Lertkiattrakul *et al.*, 2023) for ETYSUC I (blue), ETYSUC all data (orange) and ETYFUM (red). (Patyk-Kaźmierczak & Kaźmierczak, 2024).

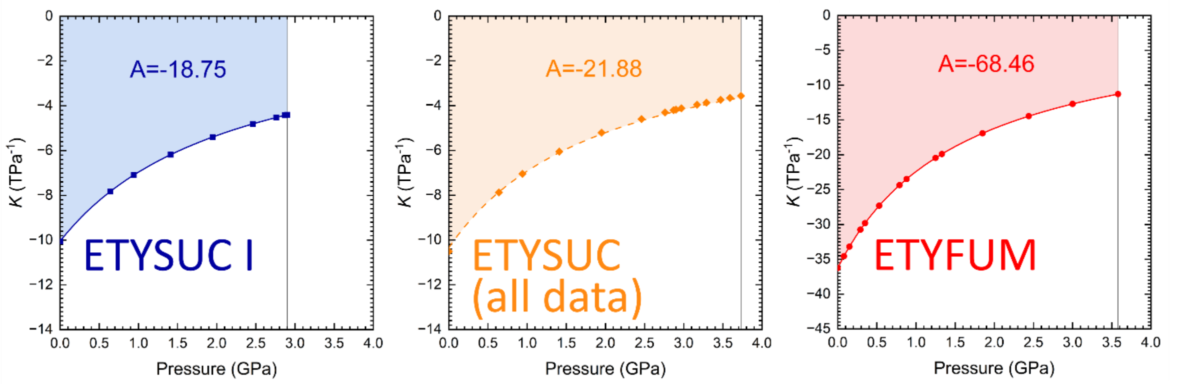

Figure S13 Pressure-dependence of compressibility *K* calculated using PASCal (Cliffe & Goodwin, 2012; Lertkiattrakul *et al.*, 2023) for ETYSUC I (blue), ETYSUC all data (orange) and ETYFUM (red). (Patyk-Kaźmierczak & Kaźmierczak, 2024), with functions integrated for the complete pressure range investigated (the highlight, calculated using OriginPro 2022b). The area calculated by evaluating the integrals is expressed in [GPa·TPa^{-1}], hence it should be divided by 1000 to receive dimensionless quantity.

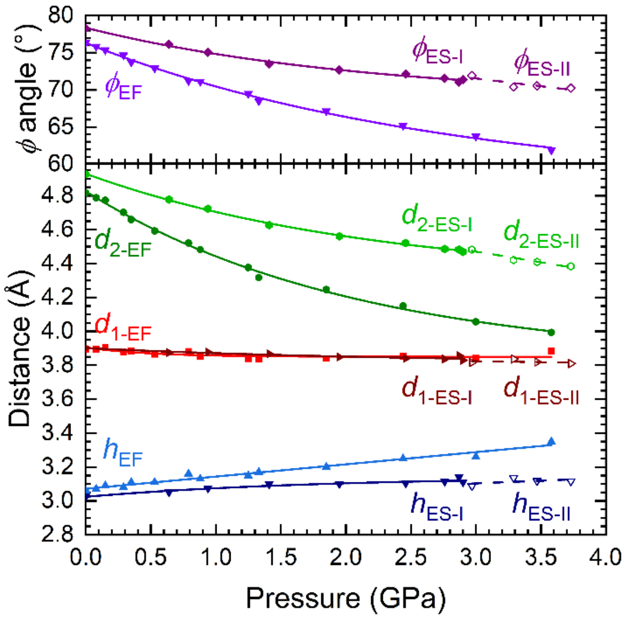

Figure S14 Pressure dependence of the parameters d_1, d_2, h and φ of a triangle formed by three adjacent *hinge points* in ETYFUM (solid red, green, blue, purple lines, respectively), ETYSUC I (solid dark red, light green, navy blue, dark purple lines, respectively) and ETYSUC I' (dashed dark red, light green, navy blue, dark purple lines, respectively). Solid lines show functions $y = a - bc^x$(where x is pressure in GPa) fitted to parameters determined based on experimentally determined structures (ETYSUC I: Tables S32-S33; ETYFUM: cited after (Patyk-Kaźmierczak & Kaźmierczak, 2024). Dotted lines represent functions $y = a + bx$ (where x is pressure in GPa) fitted to parameters determined based on experimentally determined structures of ETYSUC I' (Tables S32-S33).

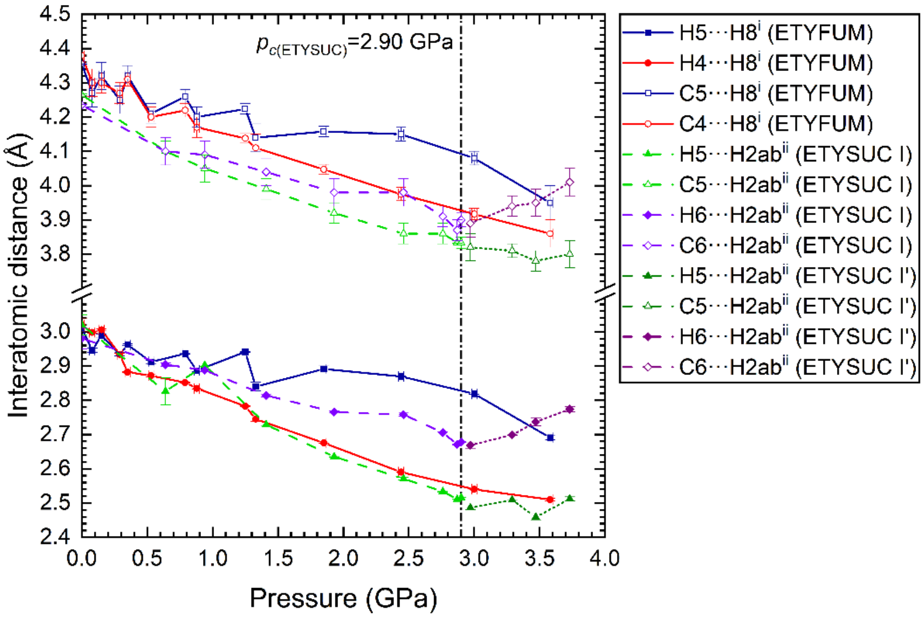

Figure S15 Pressure dependence of the interatomic H···H and C···H distances between ETY and SUC/FUM molecules in structures of ETYSUC/ETYFUM. Symmetry codes: (i) -*x*, -½+*y*, ½-*z*; (ii) ½+*x*, -*y*, *z*. The vertical dashed-dotted line marks phase transition pressure for ETYSUC.

S3.6. Thermal expansion

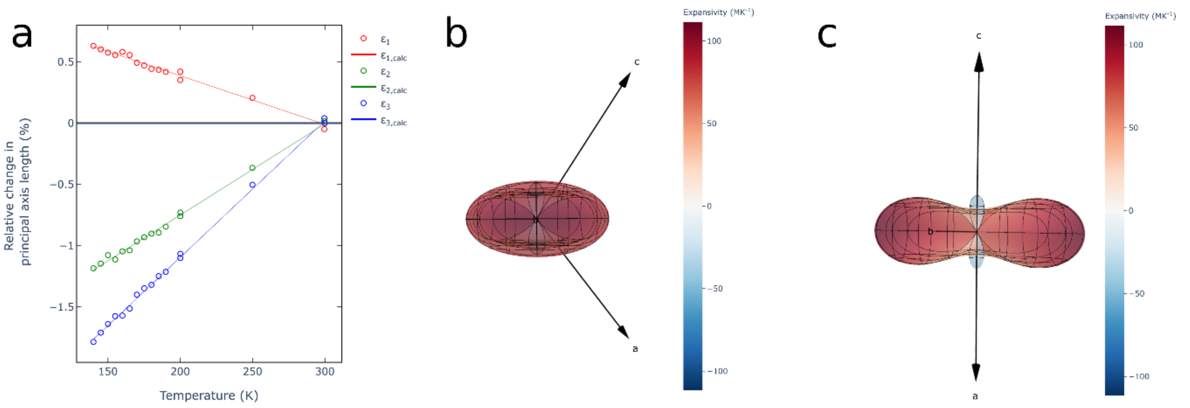

Figure S16 Relative change in length of the principal axes in function of temperature (a), and indicatrix plots representing thermal expansivity tensors (b, c) for ETYFUM (140-300 K range). The indicatrix plots are shown along crystallographic axis *b* (b), and along direction [101] (c). Positive and negative thermal expansion is marked in red and blue, respectively, in the indicatrix plots.

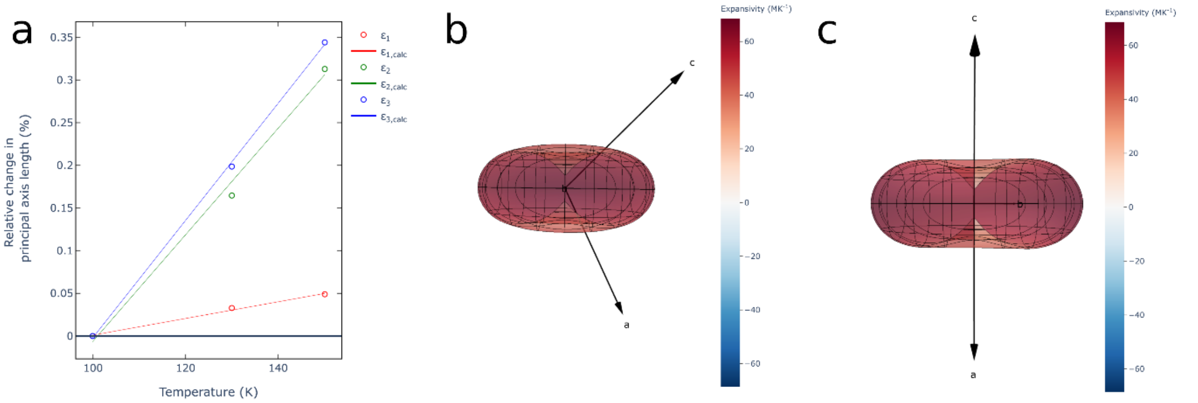

Figure S17 Relative change in length of the principal axes in function of temperature (a), and indicatrix plots representing thermal expansivity tensors (b, c) for ETYFUM (100-150 K range). The indicatrix plots are shown along crystallographic axis *b* (b), and along direction [101] (c). Positive and negative thermal expansion is marked in red and blue, respectively, in the indicatrix plots.

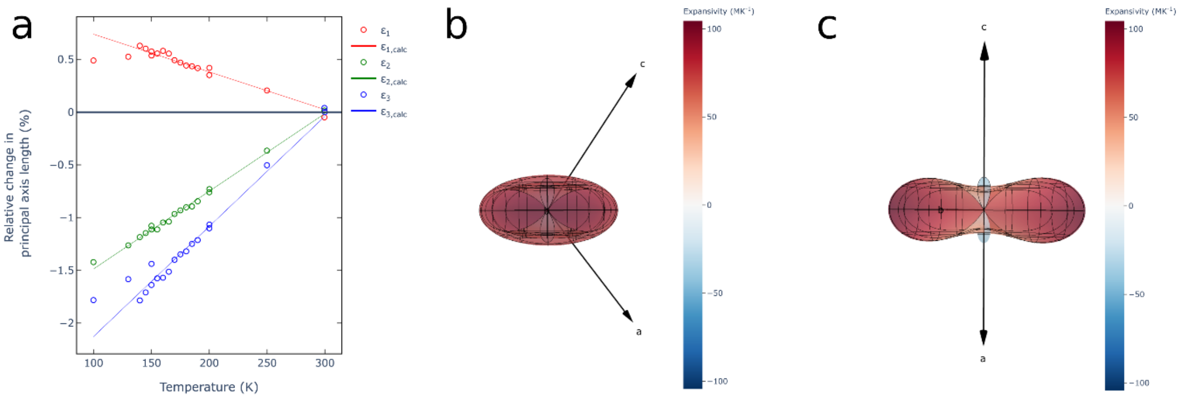

Figure S18 Relative change in length of the principal axes in function of temperature (a), and indicatrix plots representing thermal expansivity tensors (b, c) for ETYFUM (100-300 K range). The indicatrix plots are shown along crystallographic axis *b* (b), and along direction [101] (c). Positive and negative thermal expansion is marked in red and blue, respectively, in the indicatrix plots.

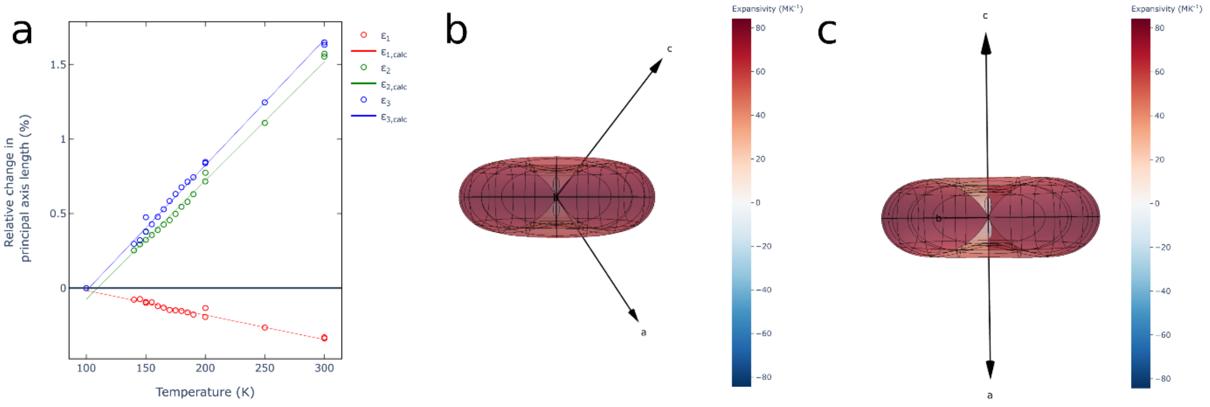

Figure S19 Relative change in length of the principal axes in function of temperature (a), and indicatrix plots representing thermal expansivity tensors (b, c) for ETYSUC (100-300 K range). The indicatrix plots are shown along crystallographic axis *b* (b), and along direction [101] (c). Positive and negative thermal expansion is marked in red and blue, respectively, in the indicatrix plots.

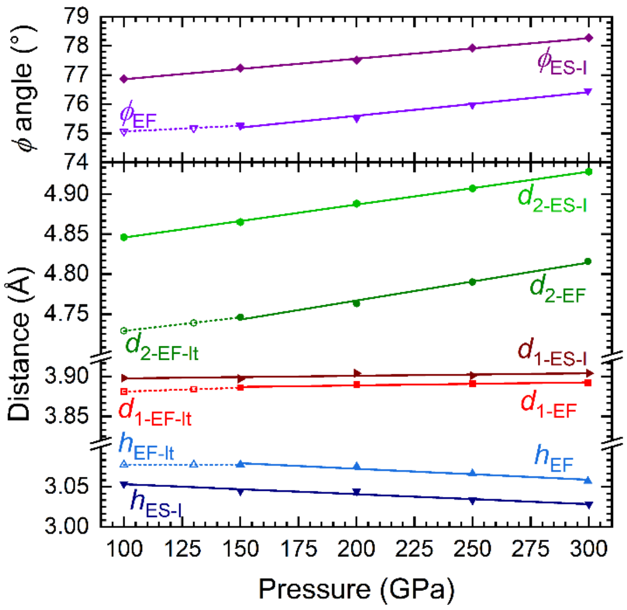

Figure S20 Temperature dependence of the parameters d_1, d_2, h and φ of a triangle formed by three adjacent *hinge points* in ETYFUM 150-300 K range (solid red, green, blue, purple lines, respectively), ETYFUM 100-150 K range (dotted red, green, blue, purple lines, respectively), ETYSUC I (solid dark red, light green, navy blue, dark purple lines, respectively). Solid and dotted lines show functions $y=a+bx$ (where x is pressure in GPa) fitted to parameters determined based on experimentally determined structures (Tables S34-S37).

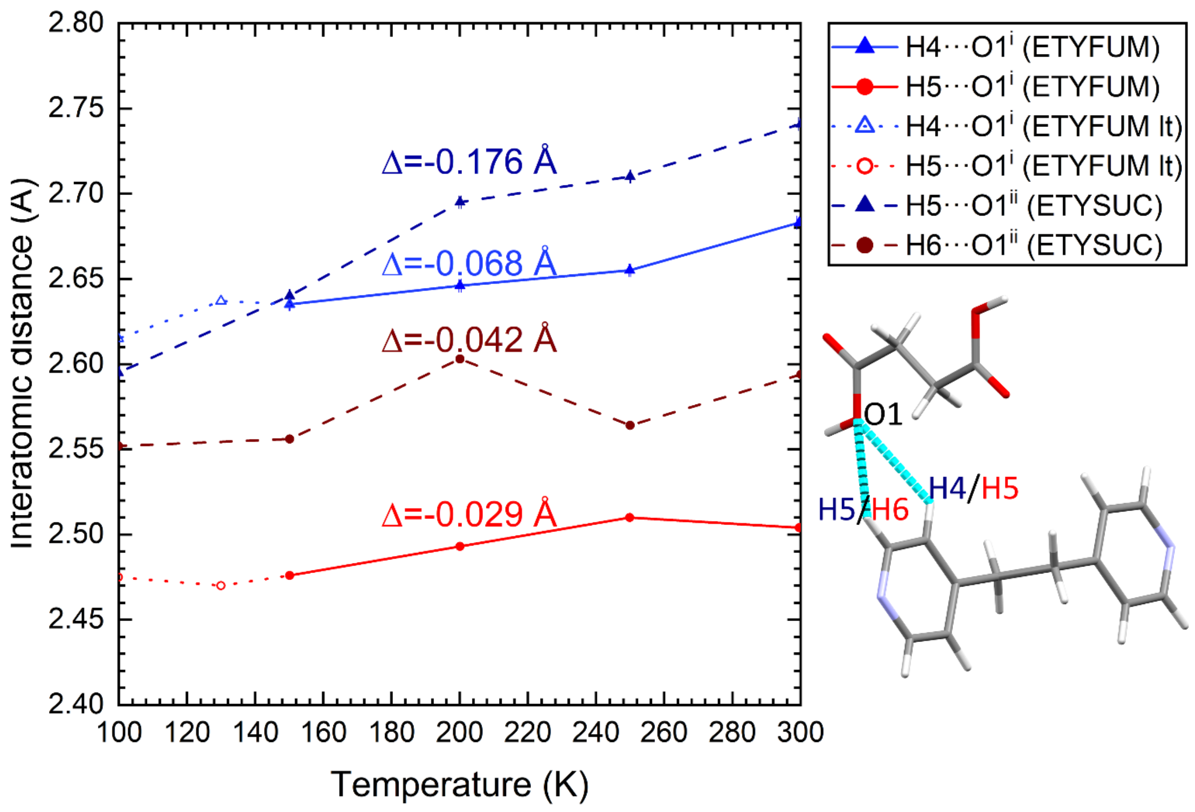

Figure S21 Temperature dependence of the length of C-H···O bonds at *hinge point* in ETYFUM (solid lines) and ETYSUC (dashed lines). Difference between the length at 300 and 100 K (Δ) is included for each bond next to the respective plot. An insert shows SUC and ETY molecules connected *via* C-H···O bonds that are plotted in the graph (analogous arrangement is present in structure of ETYFUM). The H-atoms labels in blue are for ETYFUM structure and red for ETYSUC. The symmetry codes: (i) -*x*, -½+*y*, ½-*z*; (ii) ½+*x*, -*y*, *z*.

S3.7. Pressure- and temperature-induced conformational changes

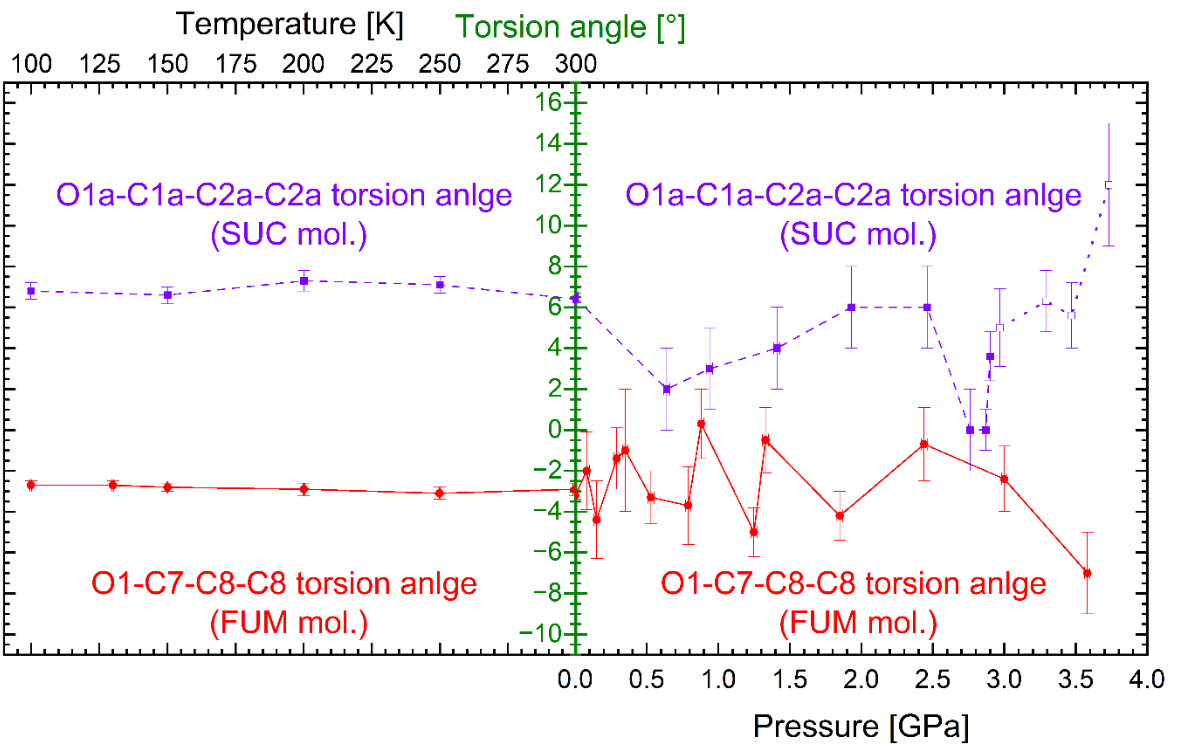

Figure S22 Pressure (right) and temperature (left) induced changes in O1a-C1a-C2a-C2a (purple) and O1-C7-C8-C8 (red) torsion angles of SUC and FUM molecules in ETYSUC and ETYFUM crystals, respectively. Data for ETYSUC I' is shown with empty symbols and dotted lines.
